# Green and efficient iron-catalyzed cross-dehydrogenative coupling for the synthesis of α,β-unsaturated ketones *via* C(sp^3^)–H functionalization†

**DOI:** 10.1039/d5ra01979f

**Published:** 2025-06-18

**Authors:** Manjit Singh, Poonam Rajesh Prasad

**Affiliations:** a Department of Chemistry, Institute of Science, Banaras Hindu University Varanasi UP 221005 India manjitsingh.rs.chy19@itbhu.ac.in poonamrp.chem@bhu.ac.in

## Abstract

An efficient C–C cross-coupling approach for the synthesis of α,β-unsaturated ketones was developed through C(sp^3^)–H functionalization of acetophenone and methylarene under thermal conditions in the presence of a green catalyst, FeCl_3_·6H_2_O, with DMF as a solvent and atmospheric O_2_ (air) as an oxidant. The method was useful for a wide range of substrates, indicating good functional group compatibility and providing an innovative approach to forming new C–C bonds from inexpensive, readily available starting materials. Thus, the main advantages of the present methods are one-pot reactions, environmentally friendly approaches, cost-effectiveness, broad substrate scope, short reaction times, easy workup procedures, and good yields.

## Abstract 2

An efficient green method for synthesizing α,β-unsaturated ketones *via* C(sp^3^)–H functionalization of acetophenone and methylarene was developed using FeCl_3_·6H_2_O in DMF under air.

## Graphical abstract

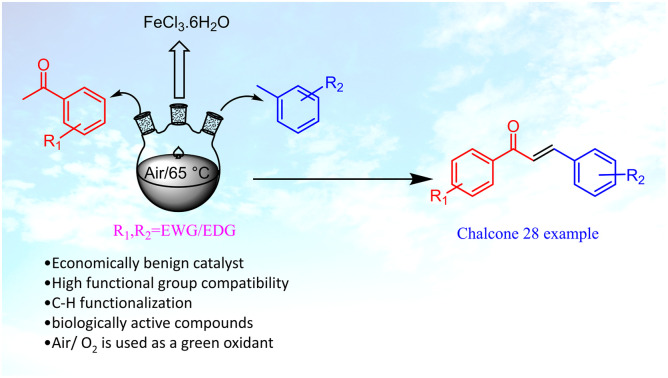

## Introduction

**Scheme 1:**
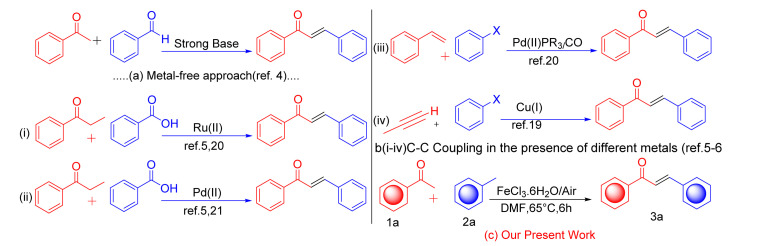
Several methods for the synthesis of *E*-chalcones.

In the new era of science, the functionalization of C(sp^3^)–H/C(sp^3^)–H bonds, typically considered inert, has attracted considerable attention as an innovative and atom-economical strategy for directly transforming simple substrates into valuable molecules. Methylarenes, in particular, play key structural roles in a wide range of organic compounds, bioactive agents, and natural products.^1^ In the field of organic chemistry, many researchers have often used methylarenes as a substrate for organic synthesis. Cross-dehydrogenative coupling (CDC) involves the introduction of an acyl group across the π-bond of an arene or acetophenone, resulting in the formation of an enone or ketone, respectively.^2^ Acetophenone plays an essential role in the formation of C–C bonds from C–H bonds.^3^ α,β-Unsaturated ketones have been widely employed in synthetic organic chemistry as versatile compounds, including as essential substrates for Michael addition, Diels–Alder, Morita–Baylis reactions, and other coupling reactions.^4^ In the field of organic chemistry, a catalyst plays a very crucial role in the formation of C–C bonds from C–H bonds. Catalysis is essential for many chemical processes, and the catalyst's qualities to improve the reactions. Many effective transition metal catalysts are used for C–C coupling reactions, such as palladium, ruthenium, and copper.^5,6^ Herein, we report iron-catalyzed cross-dehydrogenative coupling reactions between two C(sp^3^)–H bonds that interact to form C–C double bonds. Iron is the second most abundant metal in crustal abundance on Earth. Iron salt is less expensive and less toxic as compared to other metals.^7^ Its salts have limited toxicity, which has been a key factor in their use as catalysts in the food and pharmaceutical industries. Because of these benefits, its salts serve as highly effective catalysts in chemical reactions.^8,9^ In the present method, we use molecular oxygen (O_2_) (air) as the oxidizing agent. In conventional ways, a researcher commonly uses an oxidizing agent for oxidations, which is expensive and results in environmental pollution; however, herein, iron salt was oxidized by oxygen (air). The availability of air makes it very easy to use as a substitute for oxidants, thereby rendering it an ideal oxidizing agent with an eco-friendly nature and economic benefits. Synthetic chalcones display a wide range of biological activities, making them valuable for significant therapeutic advancements. They show anti-inflammatory,^10^ antibiotic,^11^ antioxidant,^12^ anticancer,^13^ antiplatelet,^14^ antidiabetic,^15^ aldose reductase-inhibitory, immunomodulatory,^16^ and non-purine xanthine oxidase-inhibitory activities. Many chalcone derivatives are used as key components of some bioactive molecules, such as metochalcone^17^ (Scheme 5), a choleretic drug. The above advantage can be seen, researchers also synthesis for chalcone in different reaction conditions such as powerful base-supported aldol condensation,^18^ palladium-catalyzed Sonogashira coupling between aryl halides and propargyl alcohols,^19^ carbonylative Heck coupling,^20^ cross-coupling of ketones with arenes or aryl carboxylic acids (Scheme 1a and b).^21^ Unfortunately, these methods suffer some drawbacks, including stoichiometric amounts of strong bases or toxic transition metal catalysts and limited functional group compatibility. To overcome this drawback, we designed a new method that fulfills the conditions of green chemistry. In this article, author report a new methodology for the synthesis of chalcone from acetophenone and toluene in the presence of the green catalyst FeCl_3_·6H_2_O and atmospheric air (Scheme 1c), which makes the high-function group compatibilities, C–H functionalization, green catalyst, biologically relevant compounds, and atmospheric oxygen is used as an ideal oxidant. Thus, the present method is accomplished with the criteria of economically and environmentally benign conditions.

## Result and discussion

A one-pot synthesis of *E*-chalcone was conducted to address reaction limitations, using stoichiometric amounts of toluene (1a, 1.0 mmol), acetophenone (2a, 1.0 mmol), and a green catalyst in a suitable solvent. This model reaction was chosen to optimize various reaction parameters, including the type of solvent, catalyst, amount of the catalyst, reaction temperature, and time, all under ambient atmospheric conditions. Firstly, we investigated the role of the solvent using various polar, nonpolar, and polar aprotic solvents (Table 1). We observed that polar protic solvents, such as water, ethanol, methanol, and isopropanol, resulted in *E*-chalcone (3a) with 0%–trace% yields (Table 1, entries 1–4). Alternatively, when we use non-polar solvents, such as toluene, hexane, and xylene, a slight enhancement was seen in the yield of the products (Table 1, entries 5–7). Similarly, we used polar aprotic solvents such as acetonitrile, 1,4-dioxane, dichloromethane, DMSO, DMF, and acetonitrile, which resulted in (3a) with 30–70% yields (Table 1, entries 8–12). Moreover, we investigated to check the next parameter—the role of the catalyst in the model reaction—using DMF as a solvent (Table 1, entries 12–14). Based on the findings, iron salt was found to be the most active catalyst, yielding product 3a in 70% yield (Table 1, entry 12). Similarly, we investigated the effect of temperature on the model reaction than the observed when temperature increases (70–100 °C) no effect on the percentage of yield thus, 65 °C is a suitable temperature for the model reaction (Table 1, entries 17–20). Next, we explored the effect of time on the model reaction. When time increased to 6–10 h (Table 1, entries 17–19) no effect on yield was observed. Thus, 6 hours was considered the optimal reaction time.

**Table 1:** The effect of the catalyst, solvent, temperature, and time on the yield of compound 3a^a^

Entry	Catalyst (mol 5%)	Solvent	Temperature (°C)	Time (h)	Yields%^b^
1	FeCl_3_·6H_2_O/air	H_2_O	65	6	NA
2	FeCl_3_·6H_2_O/air	MeOH	65	6	NA
3	FeCl_3_·6H_2_O/air	^i^PrOH	65	6	NA
4	FeCl_3_·6H_2_O/air	EtOH	65	6	Trace
5	FeCl_3_·6H_2_O/air	Toluene	65	6	30
6	FeCl_3_·6H_2_O/air	Hexane	65	6	35
7	FeCl_3_·6H_2_O/air	Xylene	65	3	40
8	FeCl_3_·6H_2_O/air	Acetonitrile	65	6	45
9	FeCl_3_·6H_2_O/air	1,4-Dioxane	65	6	40
10	FeCl_3_·6H_2_O/air	DCM	65	6	46
11	FeCl_3_·6H_2_O/air	DMSO	65	6	60
12	FeCl_3_·6H_2_O/air	DMF	65	3	70
13	Fe(OAc)_2_/air	DMF	65	6	35
14	FeBr_3_/air	DMF	65	6	50
**15**	FeCl_3_·6H_2_O/air	DMF	**65**	**6**	**84**
16	FeCl_3_·6H_2_O/air	DMF	65	6	84
17	FeCl_3_·6H_2_O/air	DMF	70	6	NR
18	FeCl_3_·6H_2_O/air	DMF	75	8	84
19	FeCl_3_·6H_2_O/air	DMF	85	10	84
20	FeCl_3_·6H_2_O/air	DMF	100	6	84

a Reaction conditions: methyl arene (1.0 mmol), acetophenone (1.20 mmol), catalyst (10 mol%), solvent (5 mL), (6 h) under open air at 65 °C temperature.

b Isolated yields.

Subsequently, the optimized reaction conditions were used to evaluate catalyst loading to improve cost-effectiveness. Several trials were conducted to reduce the amount of iron salt, and it was found that using 10 mol% of FeCl_3_·6H_2_O was sufficient to achieve a high product yield (Table 2).

**Table 2:** Investigation of the catalyst loading effect on the synthesis of compound 3a^a^

Entry	Amount of the catalyst system	Yield^b^ (%)
1	FeCl_3_·6H_2_O (5 mol%)	78
2	**FeCl**_**3**_ **·6H**_**2**_ **O (10 mol%)**	**84**
3	FeCl_3_·6H_2_O (15 mol%)	84

a Reaction conditions: methyl arene 1a (1.0 mmol), acetophenone 2a (1.0 mmol), and iron as the catalyst 3a (10 mmol).

b Isolated yields.

**Scheme 2:**
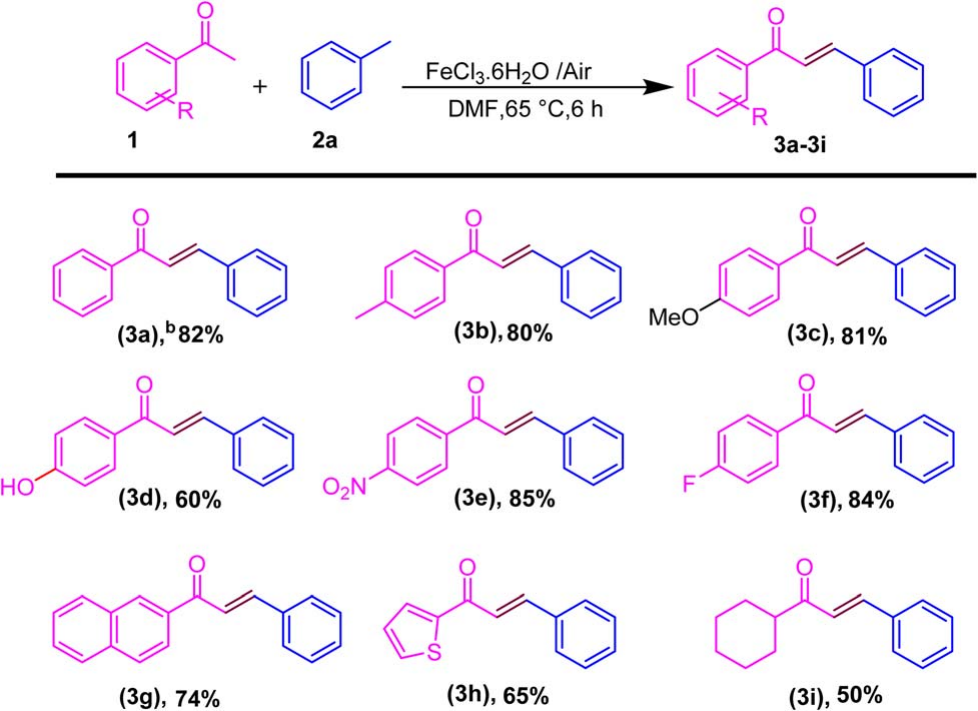
Substrate scope for the one-pot conversion of *E*-chalcones into the corresponding methyl ketone.^*a a*^Reaction conditions: all reactions were carried out with acetophenone (1) (1.0 mmol) and methyl arene (2a) (1.0 mmol) using the DMF solvent system in the presence of iron salt as the catalyst system. ^*b*^Isolated yields.

After the optimization of the reaction conditions, we next investigate the substrate scope for the synthesis of the *E*-chalcone derivatives. Substrates utilized to selectively produce (*E*)-isomers *via* hydroacylation involved acetophenones (1) and methyl arenes (2a) (Scheme 2). First, we explored a range of acetophenones and methyl arenes, and results demonstrated that the method exhibits excellent functional group compatibility. Acetophenones bearing aryl groups with varying electronic and steric properties were well tolerated, yielding the desired acylated α,β-unsaturated ketones in good to excellent yields (Scheme 2). When acetophenone contains an electron-donating group (EDG) and an electron-withdrawing group (EWG), a good to excellent yield is obtained. When methyl, methoxy, and 2-methyl are present, 80–85% yield is obtained. Alternatively, when acetophenone contains nitro and fluoro groups, 3d and 3e with 85–84% yield are obtained. Moreover, the efficiency of this transformation is largely unaffected by the steric hindrance of the aryl group (3d–3f). Disubstituted benzene rings and 1-ethynyl naphthalene effectively yielded chalcones with a 74% yield (3g). Acetophenone containing a thiophene ring produced heteroaromatic chalcones (3h) with a 65% yield, while the use of cyclohexyl-substituted acetophenone (3i) resulted in a reduced yield of 50%. Next, we focused on disubstituted acetophenones; however, no product was obtained, likely due to steric hindrance from the substituents obstructing the approach of the incoming acyl radical (see ESI, Fig. S42†). We next screened the scope of methyl arenes.

**Scheme 3:**
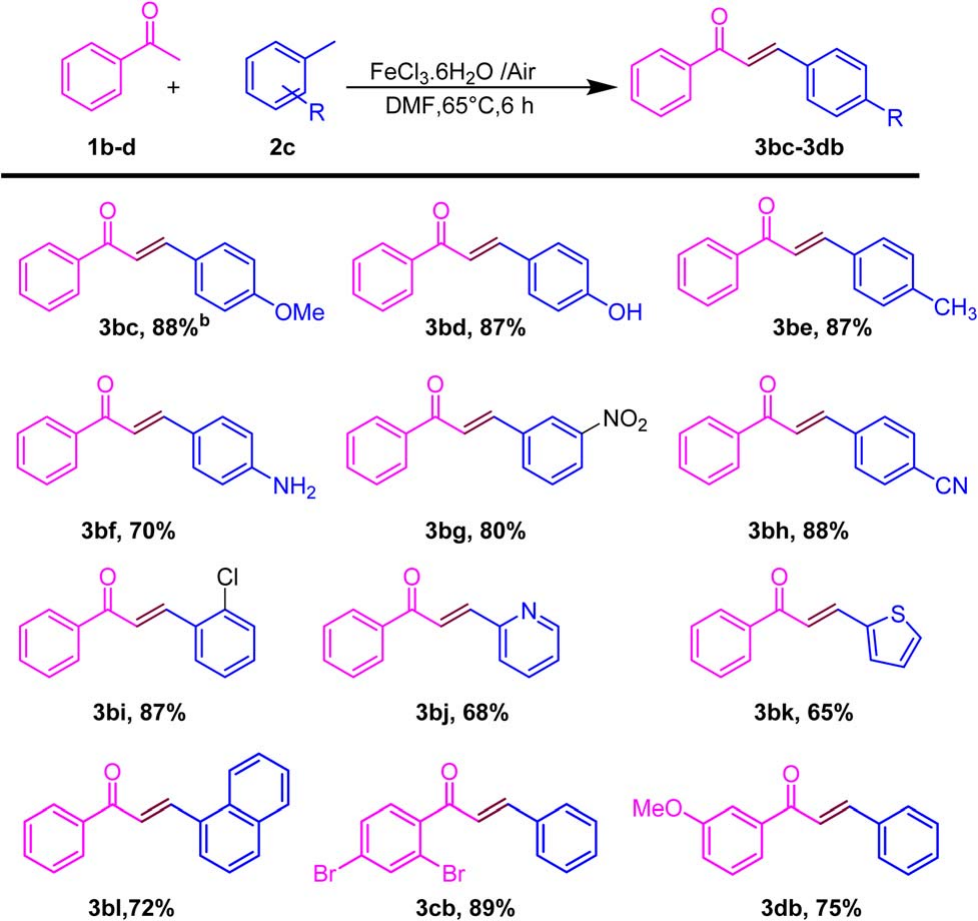
Substrate scope for the one-pot conversion of *E*-chalcone into the corresponding substituted methyl arene.^*a a*^Reaction conditions: all reactions were carried out with acetophenone (1bd) (1.0 mmol) and substituted methylarene (2c) (1.0 mmol) using the DMF solvent system in the presence of iron salt as the catalyst system. ^*b*^Isolated yields.

**Scheme 4:**
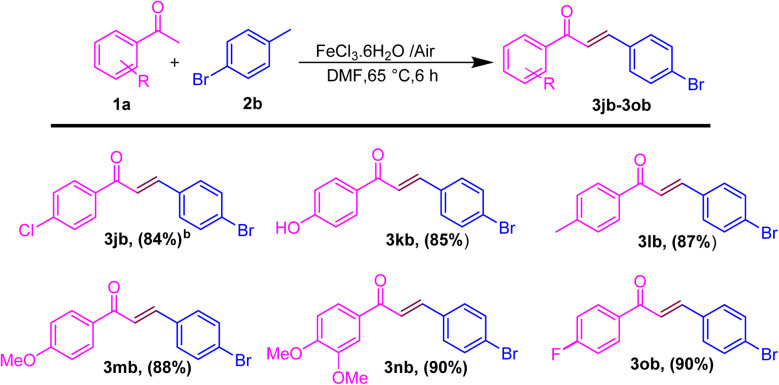
Substrate scope for the one-pot conversion of *E*-chalcone into the corresponding 4-substituted methyl ketone and 4-bromomethylarene.^*a a*^Reaction conditions: all reactions were carried out with acetophenone (1a) (1.0 mmol) and 4-bromo methylarene (2b) (1.0 mmol) using the DMF solvent system in the presence of iron salt as the catalyst system. ^*b*^Isolated yields.

**Scheme 5:**
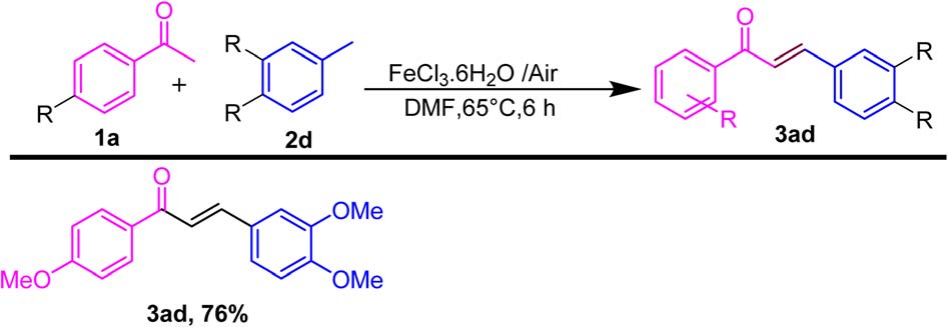
Substrate scope for the one-pot conversion of *E*-chalcone into its corresponding biologically active molecule.^*a a*^Reaction conditions: all reactions were carried out with 4-ome acetophenone (1a) (1.0 mmol) substituted with disubstituted methyl arene (2d) (1.0 mmol) using the DMF solvent system in the presence of iron salt as the catalyst system. ^*b*^Isolated yields.

After showing the universality of the acetophenone derivatives, we explored the substrate scope for the methyl arene. The formal C–C coupling reaction proceeded smoothly, producing chalcones with the required high yields, when methyl-substituted arenes were used as substrates at the *ortho*, *meta*, or *para* positions (Scheme 3). Methyl arenes bearing electron-donating groups (EDGs) such as methoxy, methyl, hydroxyl, and amino groups effectively interacted with acetophenone to generate chalcones. This approach yielded products in the range of 86–88% and 70% (3bc–3be and 3bf), respectively. The hydroacylation process proceeded efficiently with methyl arenes substituted with EWGs such as fluoro, nitro, chloro, and cyano at the *ortho*, *meta*, or *para* positions, yielding products in the range of 80–88% and 87% (3bg and 3bh; 3bi). Similarly, heteroatom substitution (3bj–3bk) and naphthalene-ring substitution (3cb–3db) provided good yield (65–72%) and disubstituted methyl arene produced the anticipated chalcone derivatives (3cb and 3db) in good (89% and 75%) yields, respectively, as shown in Scheme 3. Similarly, after the optimization of methyl arene derivatives, we further investigated the substrate scope corresponding to 4-acetophenone and 4-bromomethyl arene for obtaining good to excellent yield (3jb–3ob) (Scheme 4). Additionally, we explored a biologically active chalcone derivative (3ad) to obtain a 76% yield (Scheme 5).

**Scheme 6:**
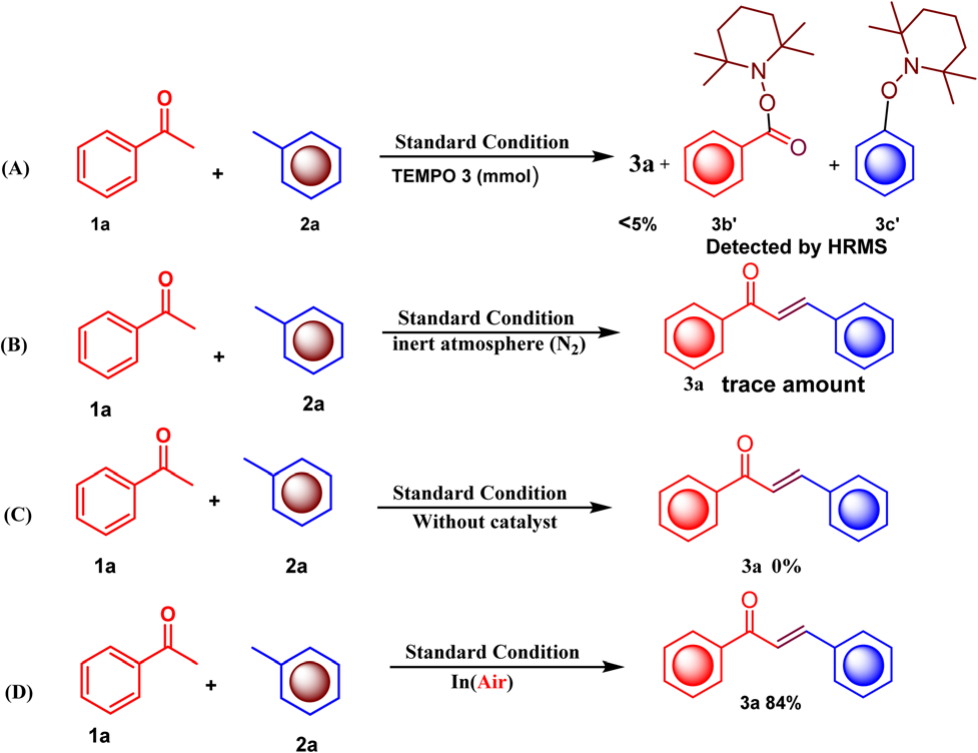
Control experiment and mechanistic investigation.

Next, we explored a controlled experiment (Scheme 6) for a mechanistic study using the radical scavenger (2,2,6,6-tetramethylpiperidin-1-yl)oxyl (TEMPO) (Scheme 6A) to investigate the reaction pathway. By maintaining the previously enhanced reaction parameters with 3 mmol of TEMPO, less than 5% of the desired product (3a) was obtained. This observation shows that the reaction proceeds through the radical pathway, which was confirmed using HRMS spectra. The next control reaction was performed between methyl arene 1a (1.0 mmol) and acetophenone 2a (1.0 mmol). The reaction was conducted under an inert atmosphere (N_2_), resulting in only a trace amount of 3a (Scheme 6B). This result signifies the importance of air (molecular oxygen). Air can be considered diluted oxygen, which helps the oxidation of Fe(ii) to Fe(iii).^22^ However, the next control reaction was performed without a catalyst, which failed to give the product (Scheme 6C). This indicates that acetophenone is not converted into an acetophenone radical intermediate without iron salt as a catalyst in the presence of air and DMF as a promoting medium. The same reaction was carried out in the presence of iron salt as a catalyst to provide the desired product in 84% yield (Scheme 6D). These results indicate that the catalyst not only participates in the oxidation of acetophenone to a α,β-unsaturated carbonyl compound through the cross-dehydrogenative coupling reaction but also acts as a coupling reagent.

### Plausible reaction mechanism

**Scheme 7:**
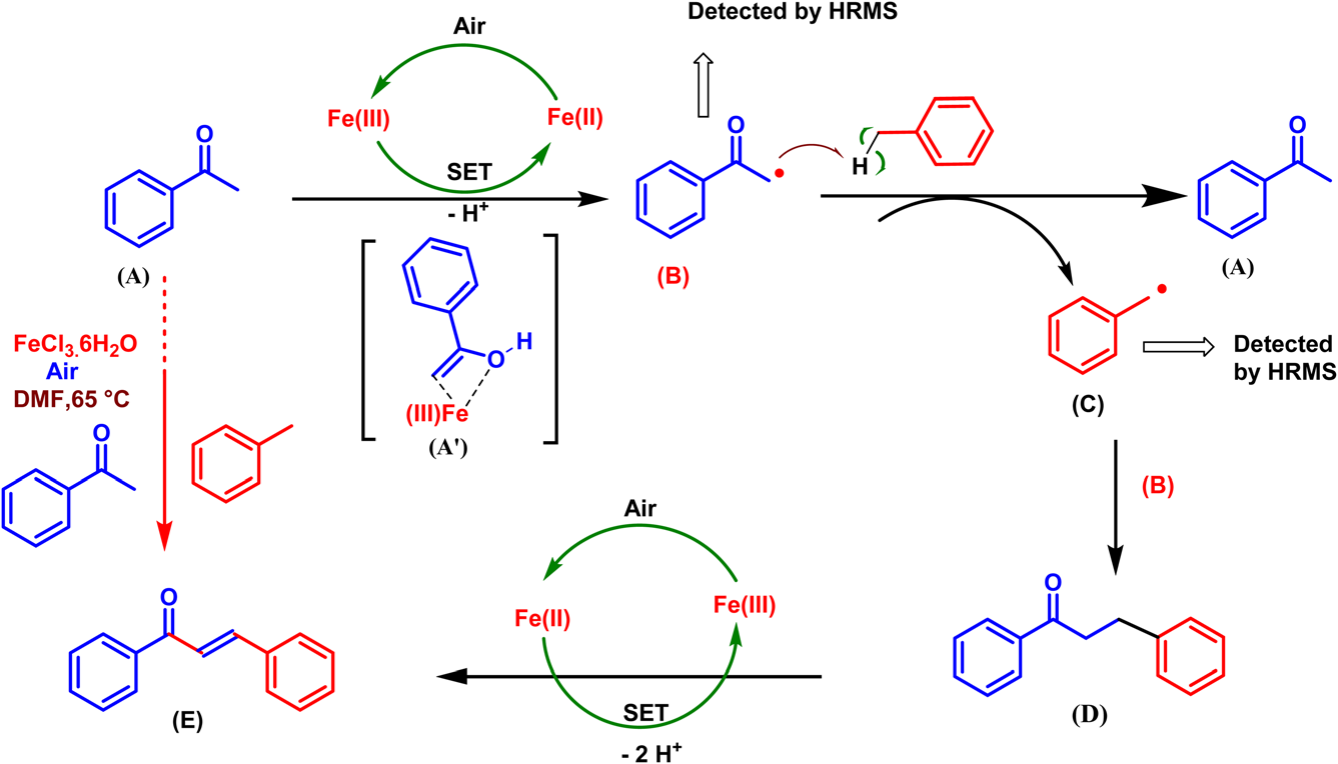
Plausible reaction mechanism for the synthesis of *E*-chalcone (E).

On the basis of the above control experiment and previous reports,^23–30^ a plausible mechanism was proposed, as shown in Scheme 7. In the presence of air and Fe(iii), the sp^3^ carbon of acetophenone is converted into the carbon radical of acetophenone (B) *via* the single electron transfer (SET) reaction and the removal of H^+^ while simultaneously forming Fe(ii).^31^ At this stage, the substrate (A) serves as an auxiliary ligand, coordinating with Fe(iii) to form a chelated Fe complex (A′). This intermediate may play a crucial role in the oxidation process, facilitating the transformation A to B. A methyl arene radical (C) is generated when the acetophenone radical (B) removes hydrogen from methyl arene. Next, an intermediate (D) arises when the methyl arene radical (C) and acetophenone radical (B) combine,^23^ and finally, D completes the oxidation to form E^23^*via* two SETs and the loss of two H^+^.^26^

### Gram-scale synthesis of *E*-chalcone derivatives

**Scheme 8:**
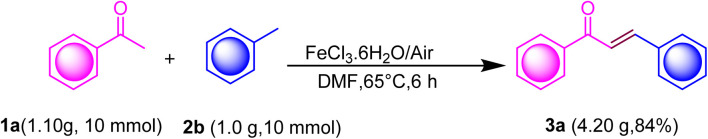
Gram-scale synthesis of *E*-chalcone.

To establish the potential synthetic application of this methodology, the synthesis of 3a was carried out on a gram scale with acetophenone (1a) (1.2 g, 10 mmol) and methylarene (2a) (1.1 g, 10 mmol) using iron salt (5.0 mg, 10 mmol) under optimized reaction conditions. Using iron salt as a catalyst, air (oxidising agent), and DMF as a promoting medium, the required products (3a) with an 84% yield (5.2 g) were obtained at 65 °C. The model reaction described in Scheme 8 was carried out on a gram scale.

## Conclusion

In conclusion, we developed a unique method for the C–C coupling reaction of acetophenone C(sp^3^)–H functionalized methyl arenes as the acylating agent using Fe(iii) catalysis with DMF as a solvent. This cross-dehydrogenative coupling (CDC) reaction allows various chalcones to be smoothly formed in good to excellent yields with elevated tolerance for functional groups. In the current approach, we used iron as an economical, safe catalyst and atmospheric air as an oxidant in place of chemical oxidants (H_2_O_2,_ Na_2_S_2_O_6_, and KMnO_4_, *etc.*), which make it cost-effective, atom-efficient, and ecologically benign and tolerate various functional groups.

## Author contributions

Manjit Singh performed all the experiments and manuscript writing as well as constructed all the figures; Poonam Rajesh Prasad provided valuable suggestions during the experimental work and reviewed the whole manuscript.

## Conflicts of interest

The authors declare no competing financial interests.

## Supplementary Material

RA-015-D5RA01979F-s001

## Data Availability

The data underlying this study are available in the published article and its ESI.†
