# Supplementary material for: Green and efficient iron-catalyzed cross-dehydrogenative coupling for the synthesis of α,β-unsaturated ketones via C(sp3)–H functionalization
Source: RSC Adv. 2025 Jun 18;15(26):20623–8. doi: 10.1039/d5ra01979f (PMC12175058; doi:10.1039/d5ra01979f)
Supplement: RA-015-D5RA01979F-s001 [file RA-015-D5RA01979F-s001.pdf]

Supplementary Information (SI) for RSC Advances.
This journal is © The Royal Society of Chemistry 2025

Green and Efficient Iron-catalyzed Cross-Dehydrogenative Coupling for the Synthesis of α, β-Unsaturated Ketones via C(sp^3)−H Functionalization

Manjit Singh and Poonam Rajesh Prasad*

Department of Chemistry, Institute of Science (BHU)
Varanasi, 221005 (India)
*Address for Correspondence

Dr. Ponam Rajesh Prasad, Department of Chemistry, Institute of Science Banaras Hindu University, Varanasi– 221 1

ORCID: https://orcid.org/0000-0001-7931-9652

Tel: +919362763739.

Email Id: poonamrp.chem@bhu.ac.in

LIST OF CONTESTS: page, no.

1. General Information S1
2. Experimental procedure for the synthesis of E- chalcone S2
3. Analytical data of products S3
4. ^{1}H, ^{13}C NMR & Mass spectrum of products S13
5. Unsuccessful substrates scope S42
6. References S43

1.) General Information

All chemicals and solvents are purchased from Sigma Aldrich and used without purification. Melting points were measured on the Stewart melting point apparatus in one side open capillary and are uncorrected. The progress of the reaction was monitored by thin-layer chromatography on glass plate coated with silica gel G-234 and florosent silica gel. UV lamp and iodine chamber was used for the visualization of the reaction spot. High-Resolution Mass Spectrometry (HRMS) was performed using a SCIEX X500R QTOF (TOF-MS) system. ^{1}H and ^{13}C NMR spectra were recorded on Bruker Avance 500 MHz spectrometer in DMSO/$CDCl_3$ d6 using TMS as internal standard 500 MHz (^{1}H) and 126 MHz (^{13}C). All chemical shifts were reported in ppm with

reference to the DMSO peak (2.50 for ^{1}H and 39.50 for ^{13}C NMR). All coupling constants are reported in hertz (Hz). Abbreviations are, s: singlet, d: doublet, t: triplet, q: quartet, bs: broad singlet, dd: double doublet. All products synthesized were confirmed by using melting point, ^{1}H and ^{13}C NMR and comparison with the literature reports.

2.) Experimental procedures

2.1 General procedure for the synthesis of (E)-chalcone.

A mixture of methyl arenes (1.0 mmol), acetophenone (1.0 mmol), were stirred in a 50-ml round bottom flask at 65°C using $FeCl_3.6H_2O$ (10 mmol) as a catalyst in DMF and times reported in Tables 2. The progress of the reaction was monitored by TLC fallowed by the completion of the reaction. After the reaction was completed, the mixture was cooled to room temperature and crushed ice was added. The precipitate was filtered, and washed with cold water, to remove catalyst because Fe(III) is soluble in water and dried under a vacuum. The crude products were purified by recrystallization from ethanol and dried to afford the pure product. The advantages offered by this methodology are known catalyst, inexpensive, and no need for column chromatography separation for the purification of our desired product. All the products were characterized based on ^{1}H-NMR, ^{13}C-NMR and HRMS.

3. A) Analytical data for table 2 [3a-3db]

[3.1] (E)-chalcone (3a) [1]

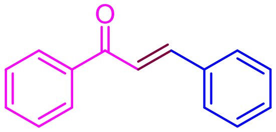

(52.1mg, 84% yield). Creamy Yellow solid. m.p.:58-60 °C. **^{1}H NMR** (500 MHz, $CDCl_3$) δ 8.23 – 8.05 (m, 2H), 7.79 (d, *J* =15.7 Hz, 1H), 7.62 (dd, *J* = 6.4, 3.2 Hz, 2H), 7.53 (t, *J* = 7.4 Hz, 1H), 7.36 – 7.27 (m, 3H), 7.23 (dd, *J* = 5.0, 1.8 Hz, 3H). **^{13}C NMR** (126 MHz,

$CDCl_3$) δ 190.5, 145.8, 137.2, 133.9, 133.8, 131.5,129.9, 129.6, 127.5, 127.4, 123.2. **HRMS** (ESI) m/z: $[M+H]^+$ calculated for $C_{15}H_{13}O$: 209.0967; found: 209.0965.

[3.2] (E)-phenyl-1-(p-tolyl) prop-2-en-1-one (3b)[1]

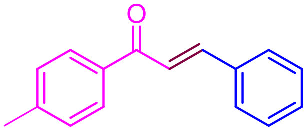

(64.0mg, 80% yield). Creamy Yellow solid. m.p.: 87-88 °C. ^{1}H NMR (500 MHz, $CDCl_3$) δ 7.81 (d, *J* =8.5 Hz, 2H), 7.80 (d, *J* = 15.7 Hz, 1H), 7.65 (d, *J* = 8.5 Hz, 2H), 7.59 (d, *J* = 8.1 Hz, 2H), 7.44 (d,*J* = 15.7 Hz, 1H), 7.25 (d, *J* = 7.9 Hz, 2H), 2.43 (s, 3H). ^{13}C NMR (126 MHz, $CDCl_3$) δ 188.5, 144.5, 140.4, 138.1, 130.9, 131.0, 128.7, 127.5, 126.7, 121.5, 20.5. HRMS (ESI) m/z: $[M+H]^+$calculated for $C_{16}H_{14}O$: 222.0228; found: 222.0225.

[3.3] (E)-1-(4-methoxyphenyl)-3-phenylprop-2-en-1-one (3c)[2]

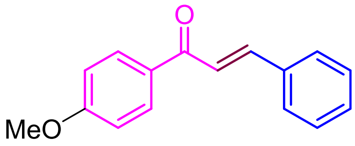

(51.8mg, 81% yield). Creamy Yellow solid. m.p.: 80-82 °C. **^{1}H NMR** (500 MHz, DMSO-d6) δ 8.17 (d, *J* = 8.9Hz, 2H), 7.96 (d, *J* = 15.6 Hz, 1H), 7.88 (dd, *J* = 7.2, 2.2 Hz, 2H), 7.73 (d, *J* = 15.6 Hz, 1H), 7.45(d, *J* = 1.7 Hz, 3H), 7.11 (d, *J* = 8.9 Hz, 2H), 3.89 (s, 3H). **^{13}C NMR** (126 MHz, DMSO-d6) δ 188.8, 164.7, 145.6, 136.3, 130.4, 131.9, 128.4, 128.3, 123.5, 115.5, 57.1. **HRMS** (ESI) m/z: $[M+H]^+$ calculated for $C_{16}H_{15}O_2$: 239.1073; found: 239.1074

[3.4] (E)-3-(4-bromophenyl)-1-(4-nitrophenyl) prop-2-en-1-one (3d)[1]

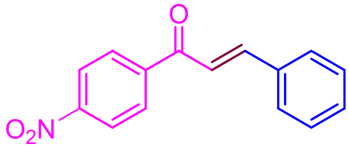

(68.0mg, 85% yield). Yellowish Brown solid. m.p.: 135-137 °C. **^{1}H NMR** (500 MHz, $CDCl_3$) δ 7.98 (d, *J* = 8.6 Hz, 2H), 7.77 (d, *J* = 15.7 Hz, 1H), 7.68 (d, *J* = 8.6 Hz, 2H), 7.61 (d, *J* = 8.4 Hz, 2H), 7.46(d, *J* = 15.7 Hz, 1H), 7.43 (d, *J* = 8.5 Hz, 2H). **^{13}C NMR**

(126 MHz, $CDCl_3$) δ 190.1, 142.9, 135.7,134.7, 132.2, 131.1, 131.0, 128.6, 128.3, 129.1, 122.9. **HRMS** (ESI) m/z: $[M+H]^+$ calculated for $C_{15}H_{11}NO_3$: 253.9923; found: 253.9925

[3.5] (E)-3-(phenyl)-1-(4-fluorophenyl) prop-2-en-1-one (3e)[3]

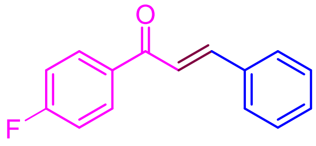

(61.7mg, 84% yield). Creamy solid solid. m.p.: 88-90 °C. **^{1}H NMR** (500 MHz, $CDCl_3$) δ 7.90 (d, J = 8.6 Hz,2H), 7.77 (d, J = 15.7 Hz, 1H), 7.67 (d, J = 8.6 Hz, 2H), 7.62 (d, J = 8.4 Hz, 2H), 7.46(d, J = 15.7Hz, 1H), 7.43 (d, J = 8.4 Hz, 2H). **^{13}C NMR** (126 MHz, CDCl3) δ 188.1, 144.8, 135.7 (d, J = 7.5Hz), 134.2, 133.0, 131.0, 128.6, 128.3, 127.1, 122.9. **^{19}F NMR** (471 MHz, $CDCl_3$) δ -115.7, -115.7, -115.8. **HRMS** (ESI) m/z: $[M+H]^+$ calculated for $C_{15}H_{11}FO$: 226.8976; found: 226.8974.

[3.6] (E)-1-(2-chlorophenyl)-3-phenylprop-2-en-1-one (3f)[3]

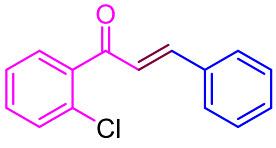

(51.4mg, 60% yield). Creamy Yellow solid. m.p.:64-66 °C. **^{1}H NMR** (500 MHz, DMSO) δ 7.77 (dd, J = 7.6, 1.7 Hz, 2H),7.62 – 7.54 (m, 3H), 7.53 (dd, J = 7.3, 1.5 Hz, 1H), 7.49 – 7.45 (m, 2H), 7.45 – 7.40 (m, 2H), 7.29(d, J = 16.1 Hz, 1H). **^{13}C NMR** (126 MHz, DMSO) δ 192.7, 147.7, 138.1, 135.5, 133.3, 130.5,131.6, 129.4, 128.7, 129.5, 128.3, 127.9, 125.7. **HRMS** (ESI) m/z: $[M+H]^+$ calculated for $C_{15}H_{11}ClO$: 242.0576; found: 242.0574.

[3.7] (E)-1-(naphthalen-1-yl)-3-phenylprop-2-en-1-one (3g)[4]

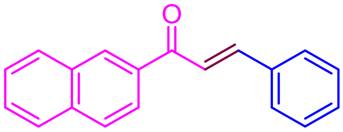

(45.8mg, 74% yield). Light Yellow solid. m.p.:187-191°C. **^{1}H NMR** (500 MHz, DMSO-d6) δ 8.71 (s, 2H), 8.25 (d, J =8.6 Hz, 4H), 8.10 (d, J = 4.6 Hz, 4H), 7.92 (dd,

J = 6.6, 3.1 Hz, 4H), 7.76(d, J = 15.6 Hz, 2H), 7.56 (d, J = 7.3 Hz, 4H), 7.47 (d, J = 5.1 Hz, 5H), 7.42 (d, J = 8.5 Hz, 4H).^{13}**C NMR** (126 MHz, DMSO-d6) δ 187.5, 155.2, 143.2, 135.2, 134.4, 133.5, 130.1, 131.5, 128.5,128.4, 123.5, 120.7. **HRMS** (ESI) m/z: $[M+H]^+$ calculated for $C_{19}H_{15}O$: 259.1125; found:259.1126.

[3.8] (E)-3-phenyl-1-(thiophen-2-yl)prop-2-en-1-one (3h)[4]

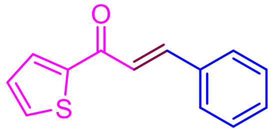
(37.4mg, 65% yield). Grey solid. m.p.: 99-100 °C.^{1}**H NMR** (500 MHz, $CDCl_3$) δ 7.92 – 7.85 (m, 2H), 7.74 (dd, J = 4.9, 1.0Hz, 1H), 7.67 (dd, J = 6.5, 3.1 Hz, 2H), 7.44 (dd, J = 8.9, 6.6 Hz, 4H), 7.24 – 7.22 (m, 1H). ^{13}**CNMR** (126 MHz, $CDCl_3$) δ 183.1, 144.5, 143.1, 133.7, 132.9, 132.8, 131.6, 127.9, 126.5, 127.2,122.7. **HRMS** (ESI) m/z: $[M+H]^+$ calculated for $C_{13}H_{10}SO$: 214.0528; found: 214.0533.

[3.9] (E)-3-cyclohexyl-1-phenylprop-2-en-1-one (3i)[5]

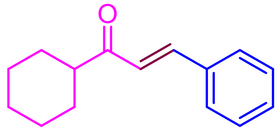
(38.4mg, 65% yield). yellowish liquid. ^{1}**H NMR** (500 MHz, $CDCl_3$) δ 7.95 (d, J = 7.7 Hz, 1H), 7.87 (d, J = 7.6 Hz, 2H), 7.47 (t, J = 7.5Hz, 2H), 6.95 (d, J = 15.1 Hz, 1H), 6.51 (dd, J = 15.1, 10.2 Hz, 1H), 3.05 – 2.98(m, 1H), 1.92 –1.80 (m, 1H), 1.05 (t, J = 7.0 Hz, 1H), 0.98 – 0.93 (m, 2H). ^{13}**C NMR** (151 MHz, $CDCl_3$) δ 187.7,153.1, 135.9, 134.0, 130.7, 130.3, 128.2, 122.7, 42.7, 41.7, 35.7, 24.9. **HRMS** (ESI) m/z: $[M+H]^+$calculated for $C_{15}H_{18}O$: 214.1358; found: 214.1255.

[3.10] (E)-1-(4-bromophenyl)-3-(4-chlorophenyl)prop-2-en-1-one (3jb)[5]

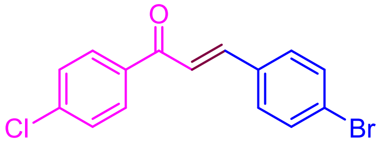

(66.5mg, 84% yield). Yellowish Brown solid. m.p.: 98-99 °C. **^{1}H NMR** (500 MHz, $CDCl_3$) δ 7.88 (d, *J* =8.5 Hz, 2H), 7.79 (d, *J* = 15.7 Hz, 1H), 7.66 (d, *J* = 8.5 Hz, 2H), 7.62 (d, *J* = 8.5 Hz, 2H), 7.44 (d,*J* = 15.7 Hz, 1H), 7.42 (d, *J* = 8.5 Hz, 2H). **^{13}C NMR** (126 MHz, $CDCl_3$) δ 187.1, 143.9, 136.7,134.7, 133.2, 132.0, 130.0, 129.6, 127.3, 126.1, 121.8. **HRMS** (ESI) m/z: $[M+H]^+$ calculated for $C_{15}H_{10}BrClO$: 319.9684; found: 320.9685.

[3.11] (E)-1-(4-bromophenyl)-3-(4-hydroxyphenyl)prop-2-en-1-one(3kb)[6]

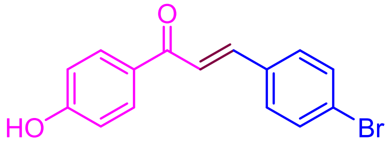

(63.2mg, 85% yield). Yellowish solid. m.p.: 82-84 °C. **^{1}H NMR** (500 MHz, $CDCl_3$) δ 9.65 (s, 1H), 7.92(d, *J* = 8.5 Hz, 2H), 7.80 (d, *J* = 15.7 Hz, 1H), 7.70 (d, *J* = 8.5 Hz, 2H), 7.65 (d, *J* = 8.5 Hz, 2H),7.45 (d, *J* = 15.7 Hz, 1H), 7.43 (d, *J* = 8.5 Hz, 2H). **^{13}C NMR** (126 MHz, $CDCl_3$) δ 190.1, 158.3,145.9, 138.7, 134.2, 130.9, 129.0, 128.6, 128.3, 127.1, 120.9. **HRMS** (ESI) m/z: [M+H] +calculated for $C_{15}H_{11}BrO_2$: 301.0010; found: 302.0025.

[3.12] (E)-1-(4-bromophenyl)-3-(p-tolyl) prop-2-en-1-one(lb)[7]

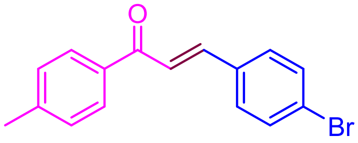

(73.2mg, 87% yield). Yellow solid. m.p.:103-104°C. **^{1}H NMR** (500 MHz, $CDCl_3$) δ 7.92 (d, *J* = 8.5 Hz, 2H),7.80 (d, *J* = 15.7 Hz, 1H), 7.65(d, *J* = 8.6 Hz, 2H), 7.58 (d, *J* = 8.1 Hz, 2H), 7.45 (d, *J* = 15.7 Hz,1H), 7.24 (d, *J* = 8.0 Hz, 2H), 2.44 (s, 3H). **^{13}C NMR** (126 MHz, $CDCl_3$) δ 190.5, 144.5, 142.4, 138.1, 130.9, 129.0, 127.8, 126.9, 126.7, 122.5, 20.6. **HRMS** (ESI) m/z: [M+H] calculated for $C_{16}H_{13}BrO$: 300.0230; found: 301.0230.

[3.13] (E)-1-(4-bromophenyl)-3-(4-methoxyphenyl) prop-2-en-1-one (3mb)[8]

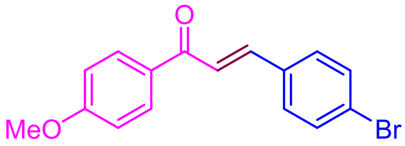

(75.2mg, 88% yield). Yellowish Brown solid. m.p.: 101-102°C. **^{1}H NMR** (500 MHz, DMSO d6) δ 8.08 (d, *J* = 8.6 Hz, 2H), 7.86 (d, *J* = 8.8 Hz, 2H), 7.79 – 7.75 (m, 4H), 7.04 (d, *J* = 8.8 Hz,2H), 3.84 (s, 3H). **^{13}C NMR** (126 MHz, DMSO-d6) δ 190.6, 165.0, 146.0, 136.3, 133.3, 130.4,131.9, 126.7, 126.5, 120.6, 115.9, 54.9. **HRMS** (ESI) m/z: [M+H] $^+$ calculated for $C_{16}H_{13}BrO_2$:317.0177; found: 318.0171.

[3.14] (E)-1-(4-bromophenyl)-3-(3,4-dimethoxyphenyl) prop-2-en-1-one (3nb)[9]

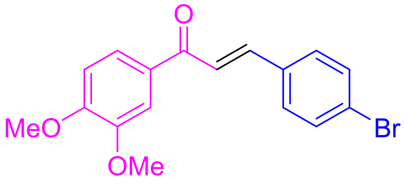

(78.2mg, 90% yield). Yellowish-brown solid. m.p.: 99-98 °C. **^{1}H NMR** (500 MHz, $CDCl_3$) δ 7.89 (d, *J* = 8.4 Hz, 2H), 7.80(d, *J* = 15.6 Hz, 1H), 7.65(d, *J* = 8.3Hz, 2H), 7.36 (d, *J* = 15.6 Hz, 1H), 7.25 (dd, *J* = 8.3, 1.9 Hz, 1H), 7.19(d, *J* = 1.8 Hz, 1H), 6.92 (d, *J* = 8.3 Hz, 1H), 3.99 (s, 3H), 3.95 (s, 3H). **^{13}C NMR** (126 MHz, $CDCl_3$) δ 190.5, 150.7, 148.3,146.6, 136.2, 130.9, 128.9, 128.7, 124.3, 118.5, 110.9, 111.2, 56.0, 55.0. **HRMS** (ESI) m/z:[M+H]+ calculated for $C_{17}H_{15}BrO_3$: 346.0285; found: 347.0286.

[3.15] (E)-3-(4-bromophenyl)-1-(4-fluorophenyl) prop-2-en-1-one (3ob)[9]

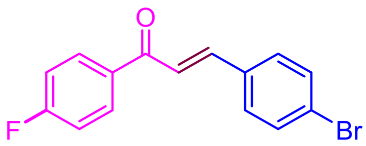

(78.7mg, 90% yield). Creamy solid solid. m.p.: 88-90°C. **^{1}H NMR** (500 MHz, $CDCl_3$) δ 7.89 (d, *J* = 8.6 Hz,2H), 7.77 (d, *J* = 15.7 Hz, 1H), 7.66 (d, *J* = 8.6 Hz, 2H), 7.66 (d, *J* = 8.4 Hz, 2H), 7.45 (d, *J* = 15.7Hz, 1H), 7.43 (d, *J* = 8.4 Hz, 2H). **^{13}C NMR** (126 MHz,

$CDCl_3$) δ 190.1, 142.8, 135.7 (d, J = 7.5 Hz), 134.2, 133.0, 129.0, 128.6, 127.3, 127.1, 122.9. **19F NMR** (471 MHz, $CDCl_3$) δ -115.7, -115.7, -115.7. **HRMS** (ESI) m/z: $[M+H]^+$ calculated for $C_{15}H_{10}BrFO$: 303.9988; found: 305.9980

[3.16] (E)-1-(4-methoxyphenyl)-3-phenylprop-2-en-1-one (3bc)[5]

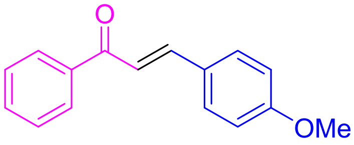

(52.8mg, 88% yield). Creamy Yellow solid. m.p.: 80-81°C. **^{1}H NMR** (500 MHz, DMSO-d6) δ 8.19 (d, *J* = 8.9 Hz, 2H), 7.94 (d, *J* = 15.6 Hz, 1H), 7.87 (dd, *J* = 7.2, 2.2 Hz, 2H), 7.70 (d, *J* = 15.6 Hz, 1H), 7.45(d, *J* = 1.7 Hz, 3H), 7.11 (d, *J* = 8.9 Hz, 2H), 3.87 (s, 3H). **^{13}C NMR** (126 MHz, DMSO-d6) δ186.8, 164.7, 145.6, 136.3, 132.4, 129.9, 128.4, 126.3, 120.5, 113.5, 55.1. **HRMS** (ESI) m/z: $[M+H]^+$ calculated for $C_{16}H_{14}O_2$: 238.1075; found: 238.1076.

[3.17] (E)-1-(4-hydroxyphenyl)-3-phenylprop-2-en-1-one (3bd)[6]

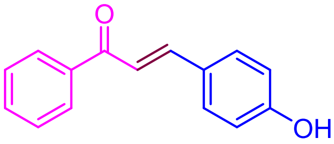

(49.0mg, 87% yield). Yellowish Brown solid. m.p.: 78-80°C. **^{1}H NMR** (500 MHz, DMSO-d6) δ 9.65 (s, 1H), 8.29(d, *J* = 8.2 Hz, 1H), 8.03 (d, *J* = 15.5 Hz, 1H), 7.85 – 7.83 (m, 2H), 7.69 – 7.64 (m, 2H), 7.44 (d, *J* = 7.1 Hz, 3H), 6.92 (d, *J* = 8.5 Hz, 1H), 6.79 (t, *J* = 7.6 Hz, 1H). **^{13}C NMR** (126 MHz, DMSO-d6) δ 195.5, 163.5, 144.7, 134.5, 130.2, 128.1, 126.2, 120.7,116.1, 115.6.**HRMS** (ESI) m/z: $[M+H]^+$calculated for $C_{15}H_{12}O_2$:224.0917; found: 224.0920.

[3.18] (E)-1-(phenyl)-3-(p-tolyl) prop-2-en-1-one (3be)[7]

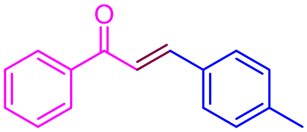

(66.7mg, 87% yield). Yellow solid. m.p.:102-103°C. **^{1}H NMR** (500 MHz, $CDCl_3$) δ 7.92 (d, *J* = 8.4 Hz, 2H),7.84 (d, *J* = 15.7 Hz, 1H), 7.65 (d, *J* = 8.6 Hz, 2H), 7.56

(d, J = 8.1 Hz, 2H), 7.45 (d, J = 15.7 Hz,1H), 7.28 (d, J = 8.0 Hz, 2H), 2.43 (s, 3H). **^{13}C NMR** (126 MHz, $CDCl_3$) δ 188.5, 144.5, 142.4, 138.1, 130.9, 131.0, 128.8, 129.9, 126.7, 121.5, 20.6. **HRMS** (ESI) m/z: $[M+H]^+$ calculated for $C_{16}H_{14}O$: 222.02229; found: 222.0230.

[3.19] (E)-3-(4-aminophenyl)-1-phenylprop-2-en-1-one (3bf)[8]

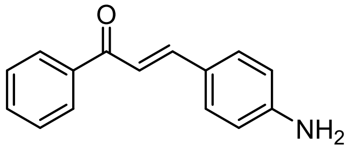

(52.6mg, 70% yield). Yellowish Brown solid. m.p.: 118-119 °C. **^{1}H NMR** (500 MHz, $CDCl_3$) δ 8.17 (d,J = 8.6 Hz, 2H), 7.79 (d, J = 15.7 Hz, 1H), 7.47 (dd, J = 12.6, 5.7 Hz, 3H), 7.21 (d, J = 7.7 Hz,1H), 6.64 (d, J = 15.7 Hz, 1H), 6.44 – 6.41 (m, 2H), 5.70 (s, 2H). **^{13}C NMR** (126 MHz, $CDCl_3$) δ188.9, 143.6, 136.6, 136.4, 135.1, 132.0, 130.4, 130.3, 129.9, 128.2, 127.9, 126.9, 122.6. **HRMS** (ESI) m/z: $[M+H]^+$ calculated for $C_{15}H_{13}NO$: 223.0180; found: 223.0185.

[3.20] (E)-3-(3-nitrophenyl)-1-phenylprop-2-en-1-one (3bg)[9]

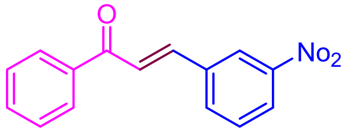

(53.8mg, 80% yield). Light Yellow solid. m.p.:116-118°C. **^{1}H NMR** (500 MHz, $CDCl_3$) δ 8.54 (s, 1H), 8.30 (dd, J = 8.2,1.4 Hz, 1H), 7.94 (d, J = 8.5 Hz, 3H), 7.87 (d, J = 15.7 Hz, 1H), 7.82 (d,J = 8.7 Hz, 1H), 7.71 (d, J = 8.6 Hz, 2H), 7.64 (dd, J = 11.8, 9.6 Hz, 2H). **^{13}C NMR** (126 MHz, $CDCl_3$) δ 188.5, 142.2, 136.5, 136.3, 134.4, 132.1, 130.1, 129.6, 128.6, 124.8, 124.0, 122.3. **HRMS** (ESI) m/z: $[M+H]^+$ calculated for $C_{15}H_{11}NO$: 253.0817; found: 254.0820.

[3.21]4-cinnamoylbenzonitrile (3bh)[10]

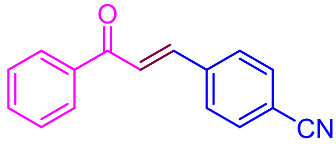 (72.5mg, 88% yield). Creamy Yellow solid. m.p.: 110-113°C. **^{1}H NMR** (500 MHz, $CDCl_3$) δ 8.12 (d, *J* = 8.6 Hz,2H), 7.84 (dd, *J* = 13.3, 12.2 Hz, 3H), 7.72 – 7.68 (m, 2H), 7.52 – 7.47 (m, 4H). **^{13}C NMR** (126MHz, $CDCl_3$) δ 190.2, 145.6, 142.5, 135.4, 133.5, 130.2, 128.1, 128.8, 127.7, 120.2, 119.0, 115.00. **HRMS** (ESI) m/z: $[M+H]^+$ calculated for $C_{16}H_{11}NO$: 234.0920; found: 234.0917.

[3.22] (E)-1-(2-chlorophenyl)-3-phenylprop-2-en-1-one (3bi)[10]

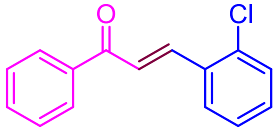 (53.4mg, 87% yield). Creamy Yellow solid. m.p.:64-66°C. **^{1}H NMR** (500 MHz, DMSO) δ 7.75 (dd, *J* = 7.6, 1.7 Hz, 2H),7.62 – 7.56 (m, 3H), 7.53 (dd, *J* = 7.3, 1.5 Hz, 1H), 7.50 – 7.46 (m, 2H), 7.44 – 7.42 (m, 2H), 7.28 (d, *J* = 16.1 Hz, 1H). **^{13}C NMR** (126 MHz, DMSO) δ 194.7, 145.7, 140.1, 135.5, 133.3, 130.5,131.6, 128.4, 127.7, 128.5, 130.3, 128.9, 125.7. **HRMS** (ESI) m/z: $[M+H]^+$ calculated for $C_{15}H_{11}ClO$: 243.0578; found: 243.0576.

[3.23] (E)-3-phenyl-1-(pyridin-2-yl)prop-2-en-1-one (3bj)[9]

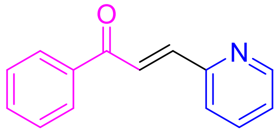 (51.4 mg, 68% yield Brown red liquid**.** **^{1}H NMR** (500 MHz, $CDCl_3$) δ 7.92 (d, *J* = 8.4 Hz, 2H), 7.77 (d, *J* = 15.6 Hz, 1H), 7.65 (d, *J* =8.3 Hz, 2H), 7.35 (d, *J* = 15.6 Hz, 1H), 7.25 (dd, *J* = 8.3, 1.9 Hz, 3H), 7.19 (d, *J* = 1.8 Hz, 1H),6.92 (d, *J* = 8.3 Hz, 1H). **^{13}C NMR** (126 MHz, $CDCl_3$) δ 190.5, 151.7, 149.3, 145.6, 131.9, 129.9,127.7, 123.3, 119.5, 111.2, 110.2. HRMS (ESI) m/z: $[M+H]^+$ calculated for $C_{14}H_{11}NO$ 210.2555; found: 210.2553.

[3.24] (E)-3-phenyl-1-(thiophen-2-yl)prop-2-en-1-one (3bk)[9]

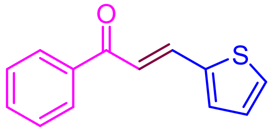 (39.4mg, 65% yield). Grey solid. m.p.: 98-99 °C.**¹H NMR** (500 MHz, $CDCl_3$) δ 7.92 – 7.85 (m, 2H), 7.72 (dd, *J* = 4.9, 1.0Hz, 1H), 7.67 (dd, *J* = 6.5, 3.1 Hz, 2H), 7.44 (dd, *J* = 8.9, 6.5 Hz, 4H), 7.24 – 7.22 (m, 1H). **¹³CNMR** (126 MHz, $CDCl_3$) δ 183.1, 144.5, 143.1, 135.7, 130.9, 130.8, 132.6, 129.9, 127.5, 127.2,120.7. **HRMS** (ESI) m/z: $[M+H]^+$ calculated for $C_{13}H_{10}SO$: 214.0538; found: 214.0531.

[3.25] (E)-1-(naphthalen-1-yl)-3-phenylprop-2-en-1-one (3bl)[5]

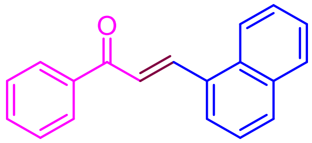 (45.8mg, 72% yield). Light Yellow solid. m.p.:187-189 °C. **¹H NMR** (500 MHz, DMSO-d6) δ 8.71 (s, 2H), 8.25 (d, *J* =8.6 Hz, 4H), 8.01 (d, *J* = 4.6 Hz, 4H), 7.93 (dd, *J* = 6.6, 3.1 Hz, 4H), 7.76 (d, *J* = 15.6 Hz, 2H), 7.55 (d, *J* = 7.3 Hz, 4H), 7.49 (d, *J* = 5.1 Hz, 5H), 7.42 (d, *J* = 8.5 Hz, 4H).**¹³C NMR** (126 MHz, DMSO-d6) δ 187.5, 155.2, 145.2, 137.2, 136.4, 131.5, 130.1, 131.5, 130.5,128.4, 123.5, 122.7. **HRMS** (ESI) m/z: $[M+H]^+$calculated for $C_{19}H_{14}O$: 258.11125; found:259.11127.

[3.26] (E)-1-(2,4-dichlorophenyl)-3-phenylprop-2-en-1-one (3cb)[4]

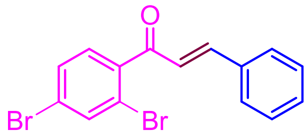 (54.7mg, 89% yield). Yellowish Brown solid m.p.: 128-129 °C. **¹H NMR** (500 MHz, DMSO) δ 8.27 (d, *J* = 8.6 Hz, 1H), 8.21 – 8.15 (m, 2H), 8.02 (q, *J* = 15.6 Hz, 2H), 7.75 (d, *J* = 2.1 Hz, 1H), 7.71 (t, *J* = 7.4 Hz,1H), 7.58 (t, *J* = 7.7 Hz, 2H), 7.55 (dd, *J* = 8.5, 2.0 Hz, 1H). **¹³C NMR** (126 MHz, DMSO) δ 190.4,138.7, 136.6, 135.1, 134.6, 134.9, 132.8, 131.3, 128.9,

127.3, 130.1, 127.4, 124.8. **HRMS** (ESI)m/z: $[M+H]^+$ calculated for $C_{15}H_{10}Br_2O$: 363.0188; found: 363.0191.

[3.27] (E)-1-(3-methoxyphenyl)-3-phenylprop-2-en-1-one (3db)[2]

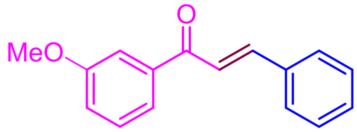

(52.4mg, 75% yield). Yellowish Brown solid. m.p.: 75-76oC. **^{1}H NMR** (500 MHz, $CDCl_3$) δ 8.06 (d, J = 8.9 Hz, 2H),7.84 (d, J = 15.7 Hz, 1H), 7.68 – 7.62 (m, 2H), 7.56 (d, J = 15.7 Hz, 1H),7.48 – 7.45 (m, 1H), 7.42 (dd, J = 4.7, 1.8 Hz, 2H), 7.00 (d, J = 8.9 Hz, 2H), 3.93 (s, 3H). **^{13}CNMR** (126 MHz, $CDCl_3$) δ 188.8, 163.5, 143.9, 135.1, 131.1, 130.8, 130.3, 128.9, 128.4, 121.9,113.8, 55.5. HRMS (ESI) m/z: $[M+H]^+$calculated for $C_{16}H_{14}O_2$:238.1082; found: 239.1076.

^{1}HNMR and ^{13}C NMR spectrum of the products

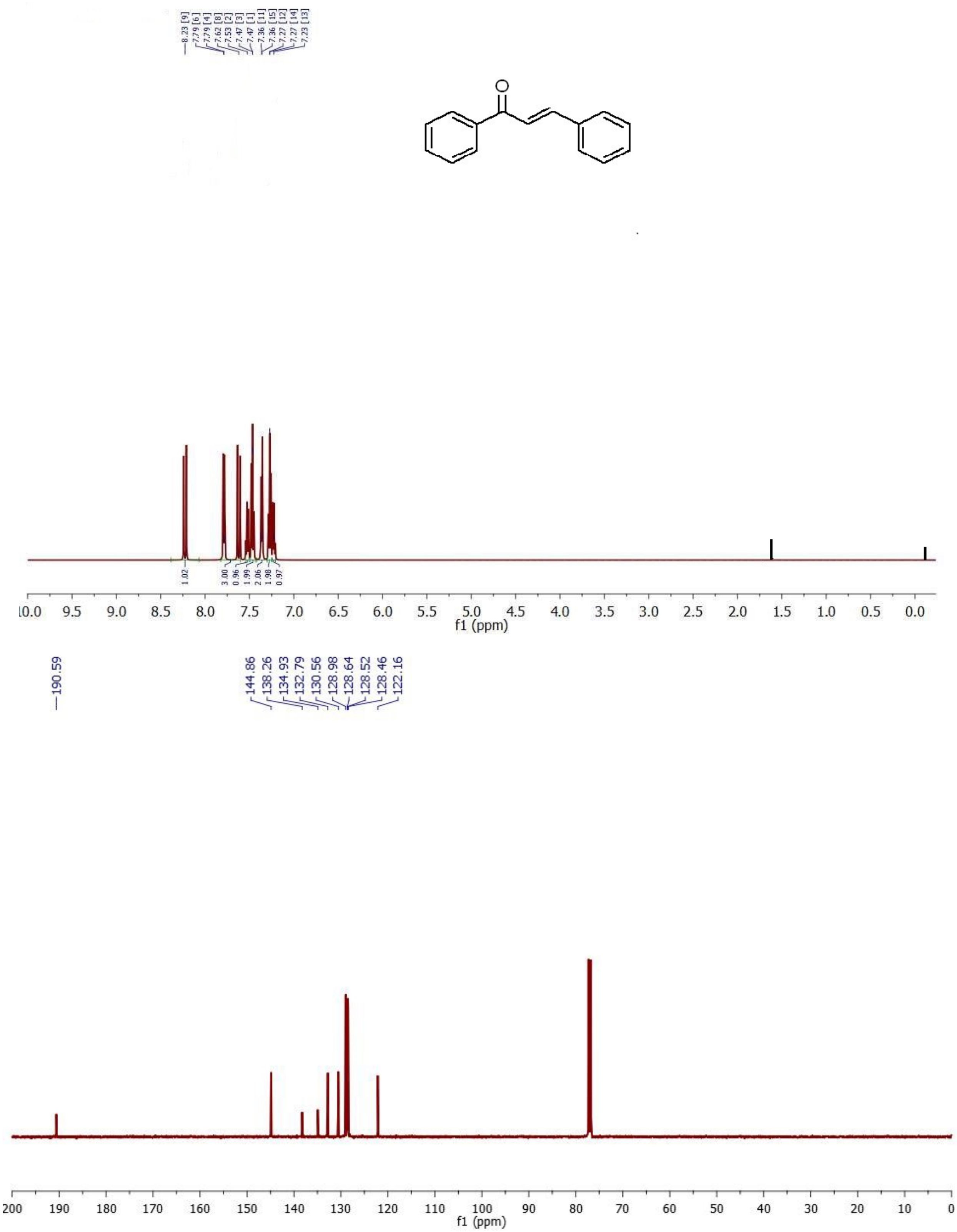

Fig.S1 ^{1}H and ^{13}C-NMR spectrum of 3a (126 MHz, $CDCl_3$)

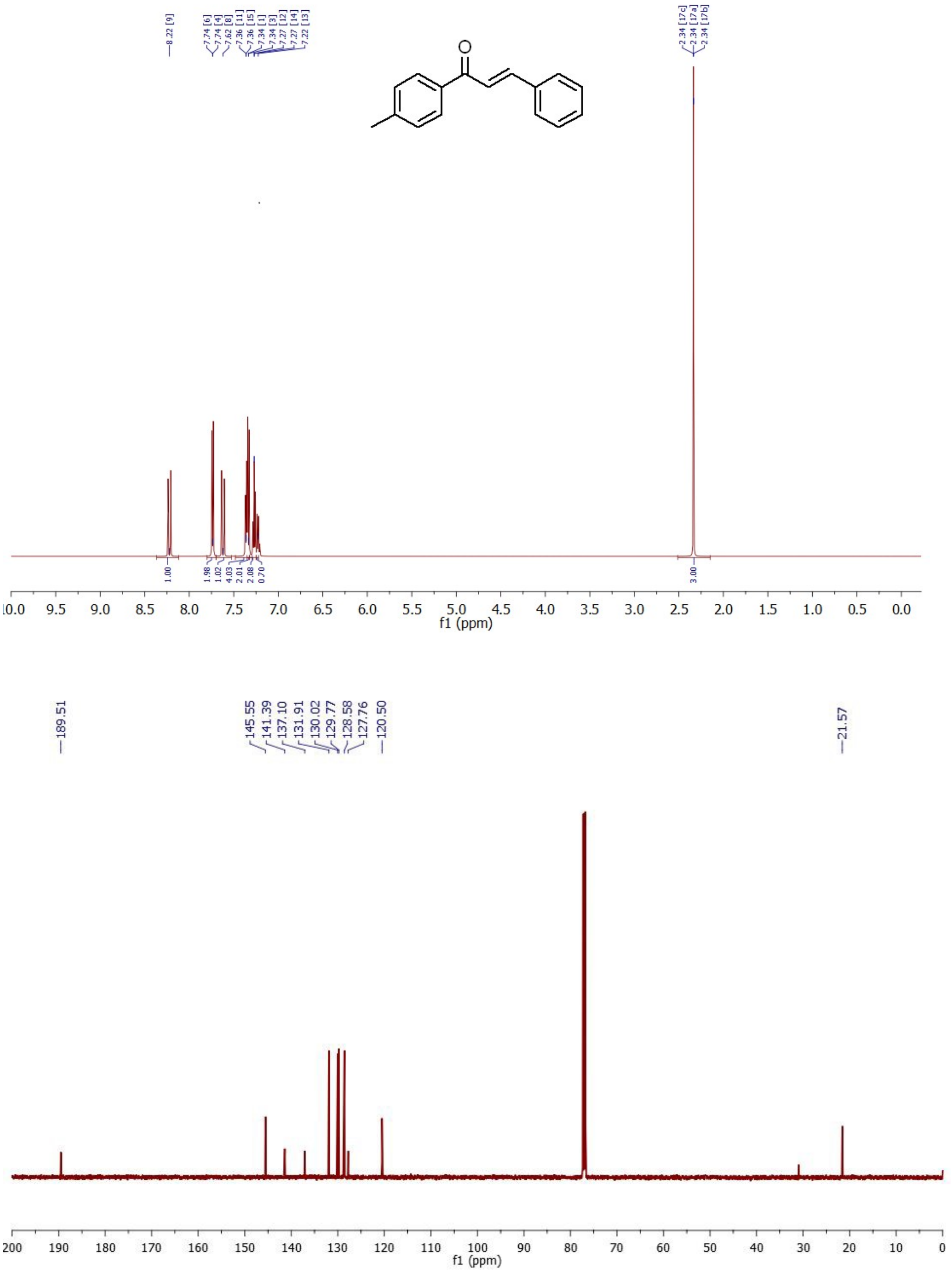

Fig.S2 ^{1}H and ^{13}C-NMR spectrum of 3b (126 MHz, $CDCl_3$)

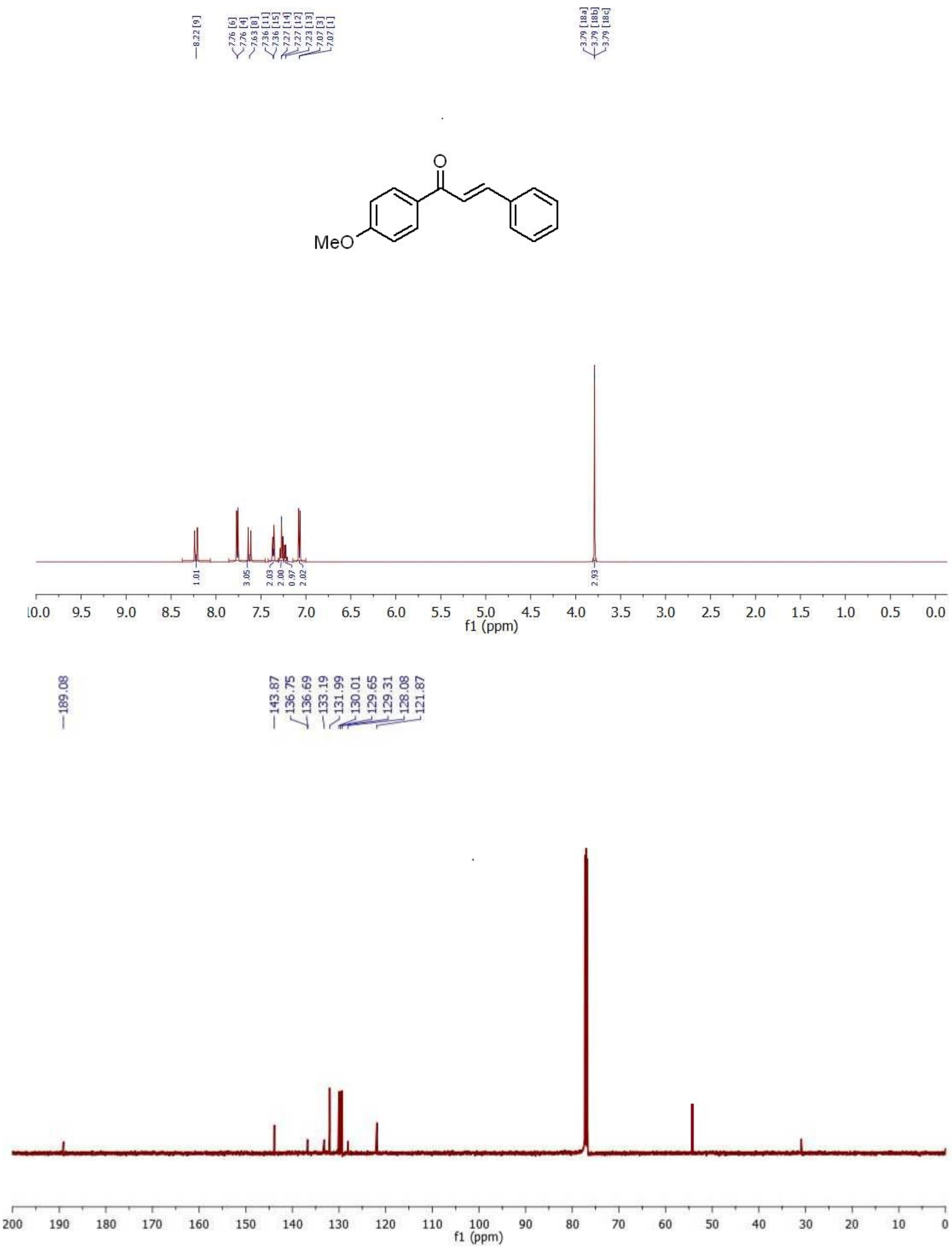

Fig.S3 ^{1}H and ^{13}C-NMR spectrum of 3c (126 MHz, $CDCl_3$)

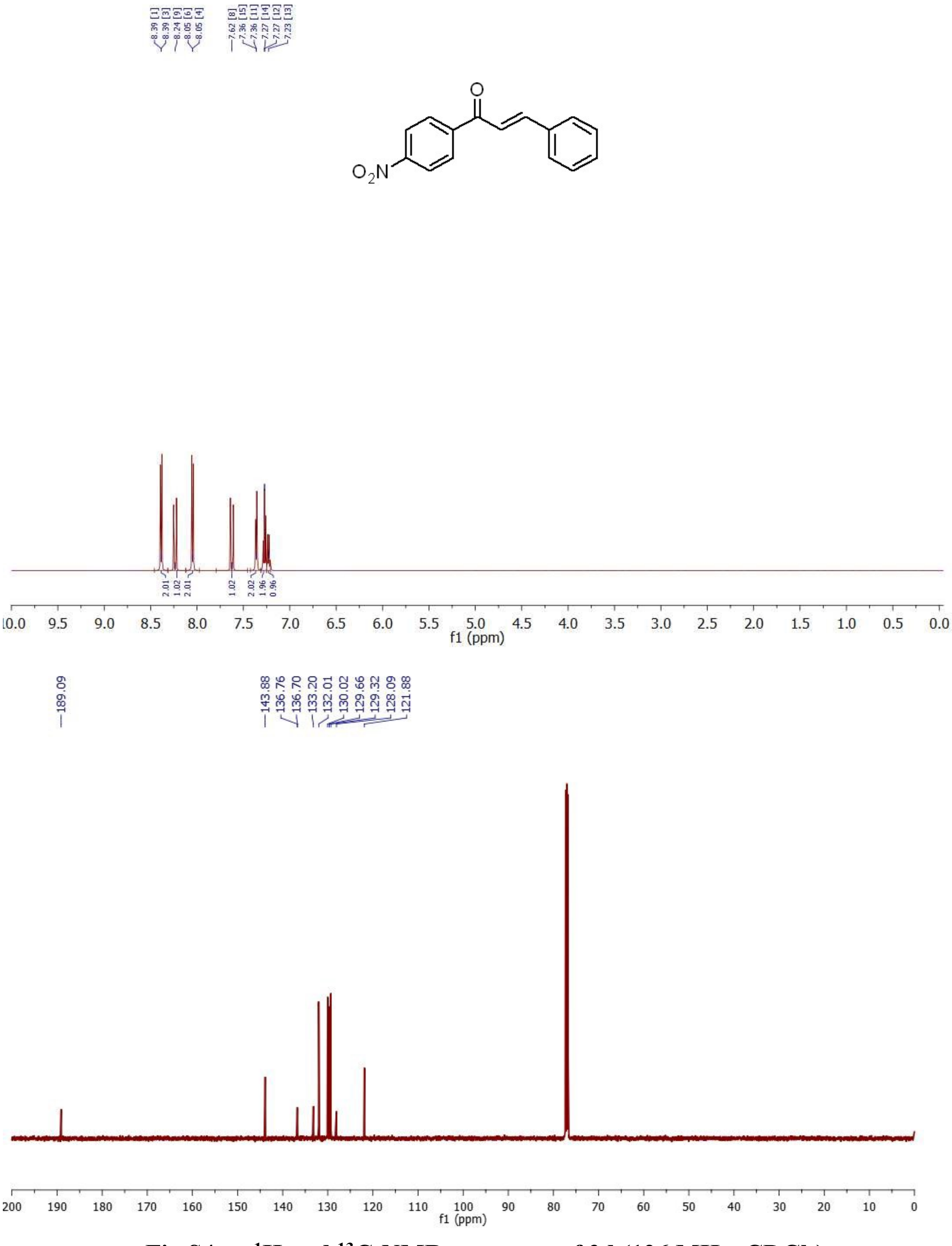

Fig.S4 ^{1}H and ^{13}C-NMR spectrum of 3d (126 MHz, $CDCl_3$)

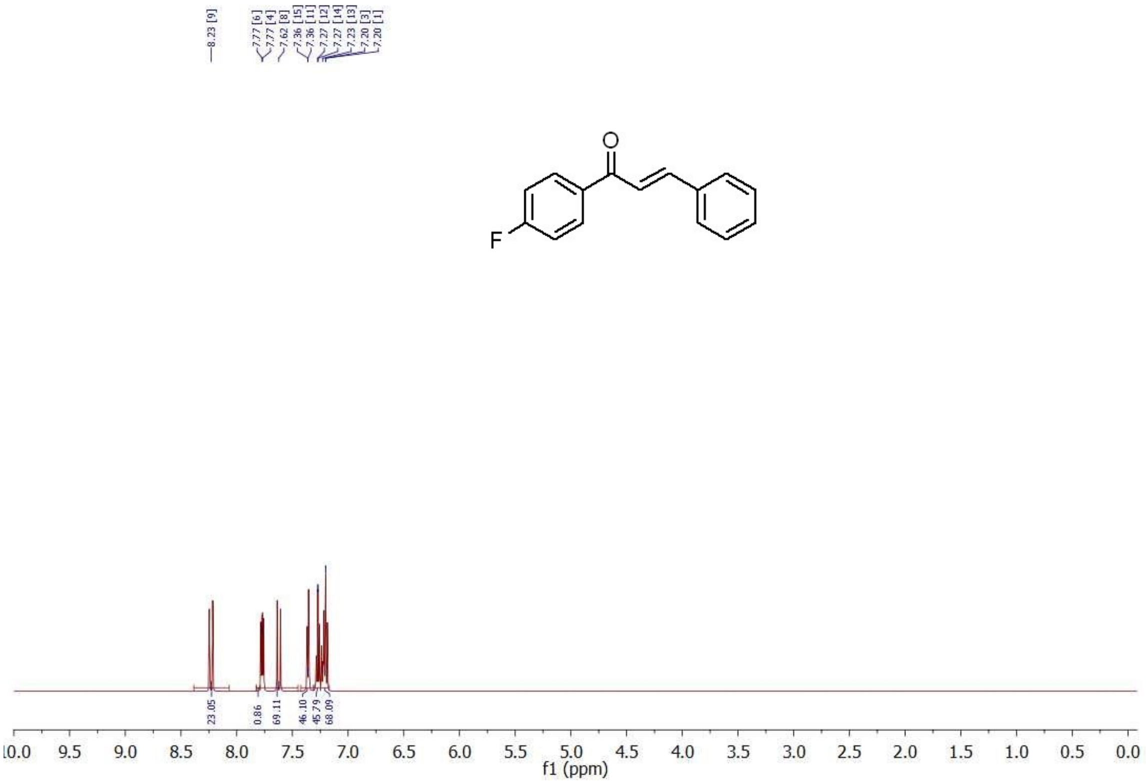

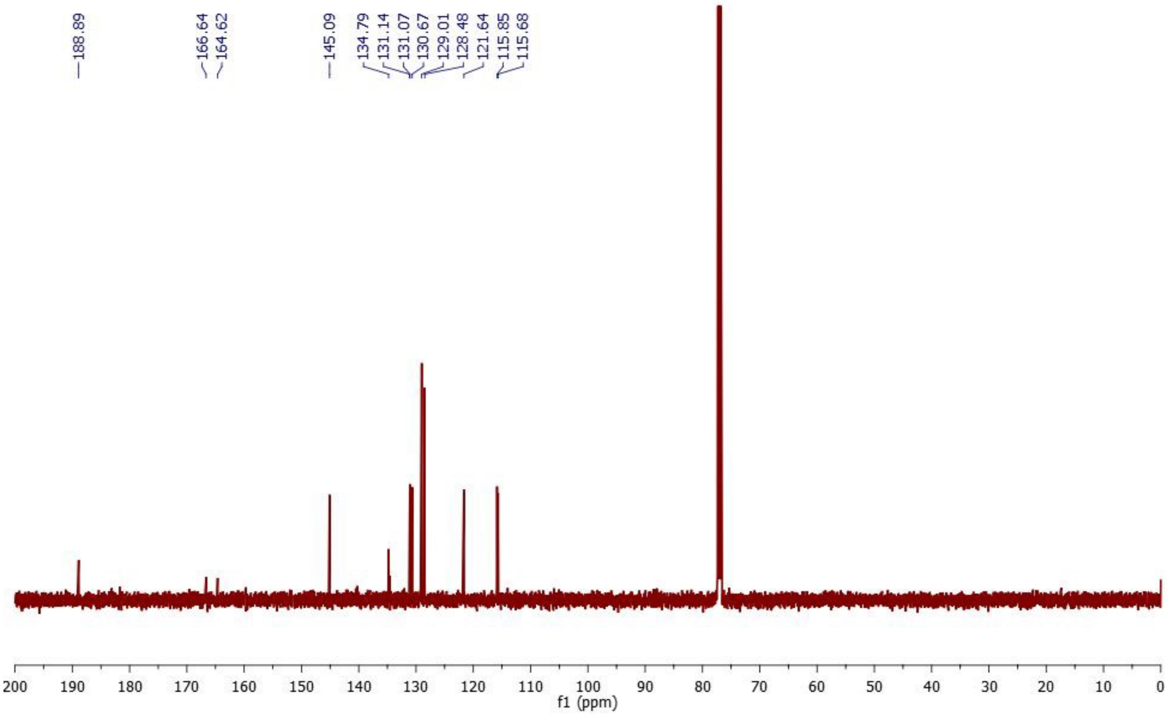

Fig.S5 ^{1}H and ^{13}C-NMR spectrum of 3e (126 MHz, $CDCl_3$)

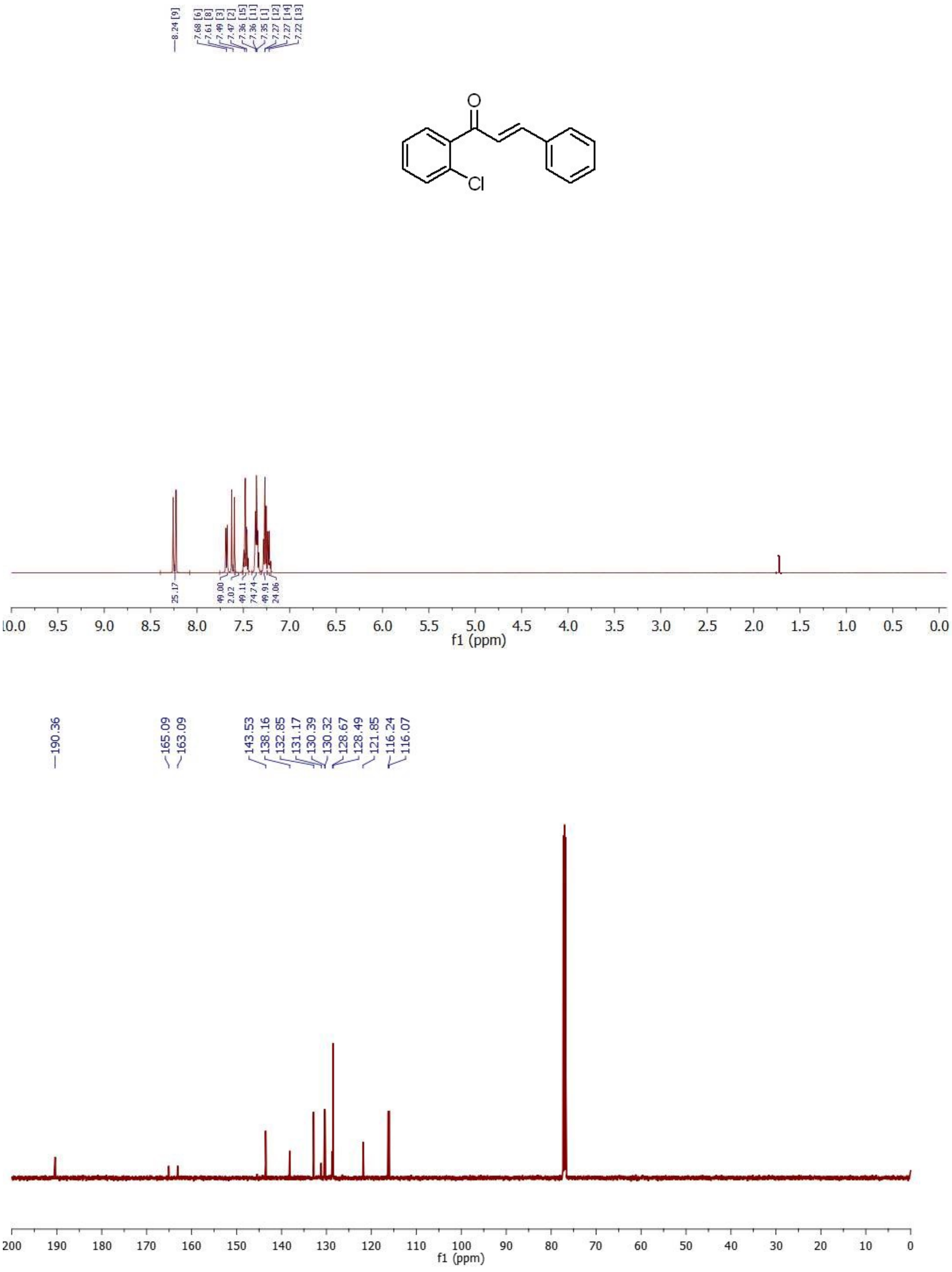

Fig.S6 ^{1}H and ^{13}C-NMR spectrum of 3f (126 MHz, $CDCl_3$)

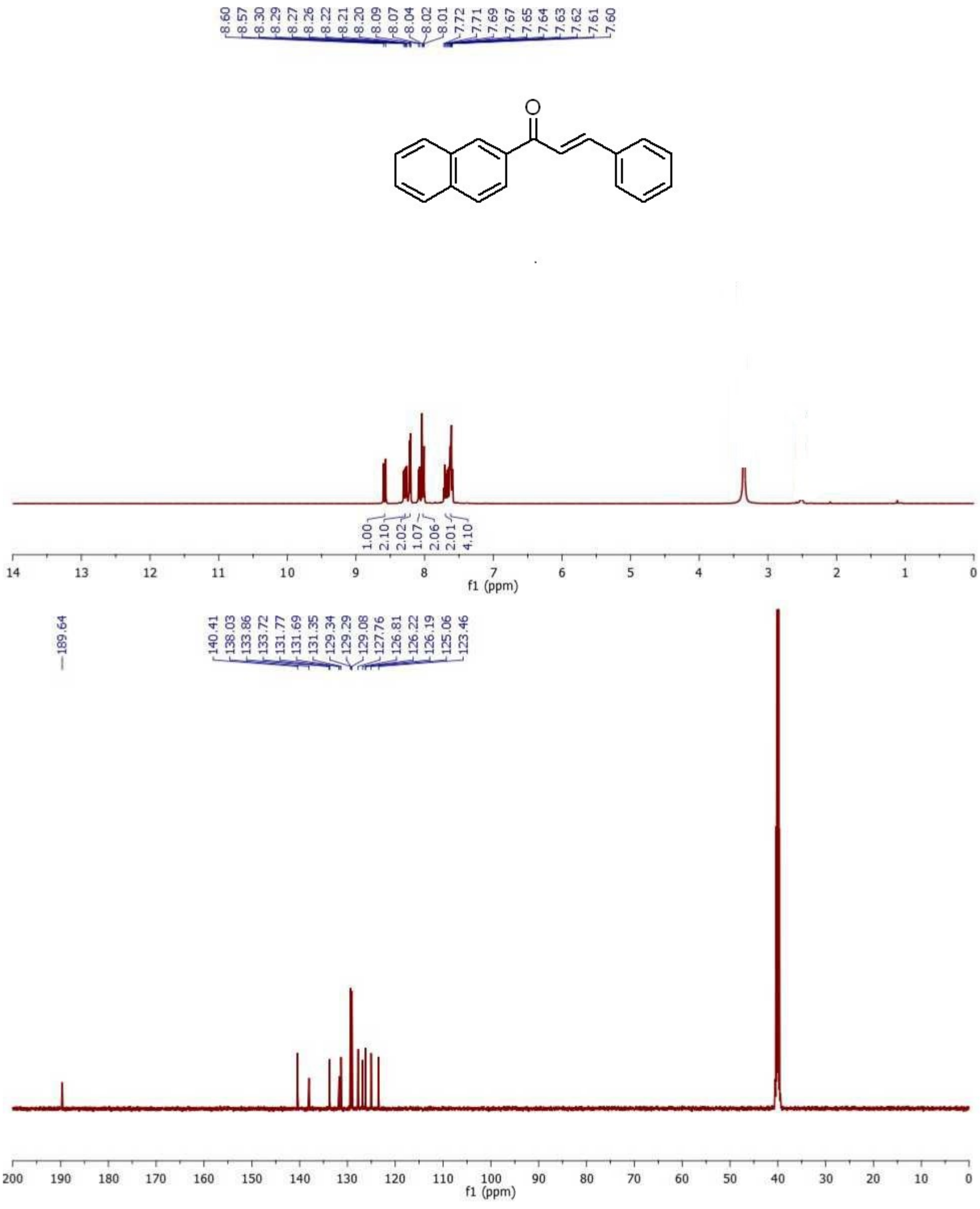

Fig.S7 ^{1}H and ^{13}C-NMR spectrum of 3g (126 MHz, $CDCl_3$)

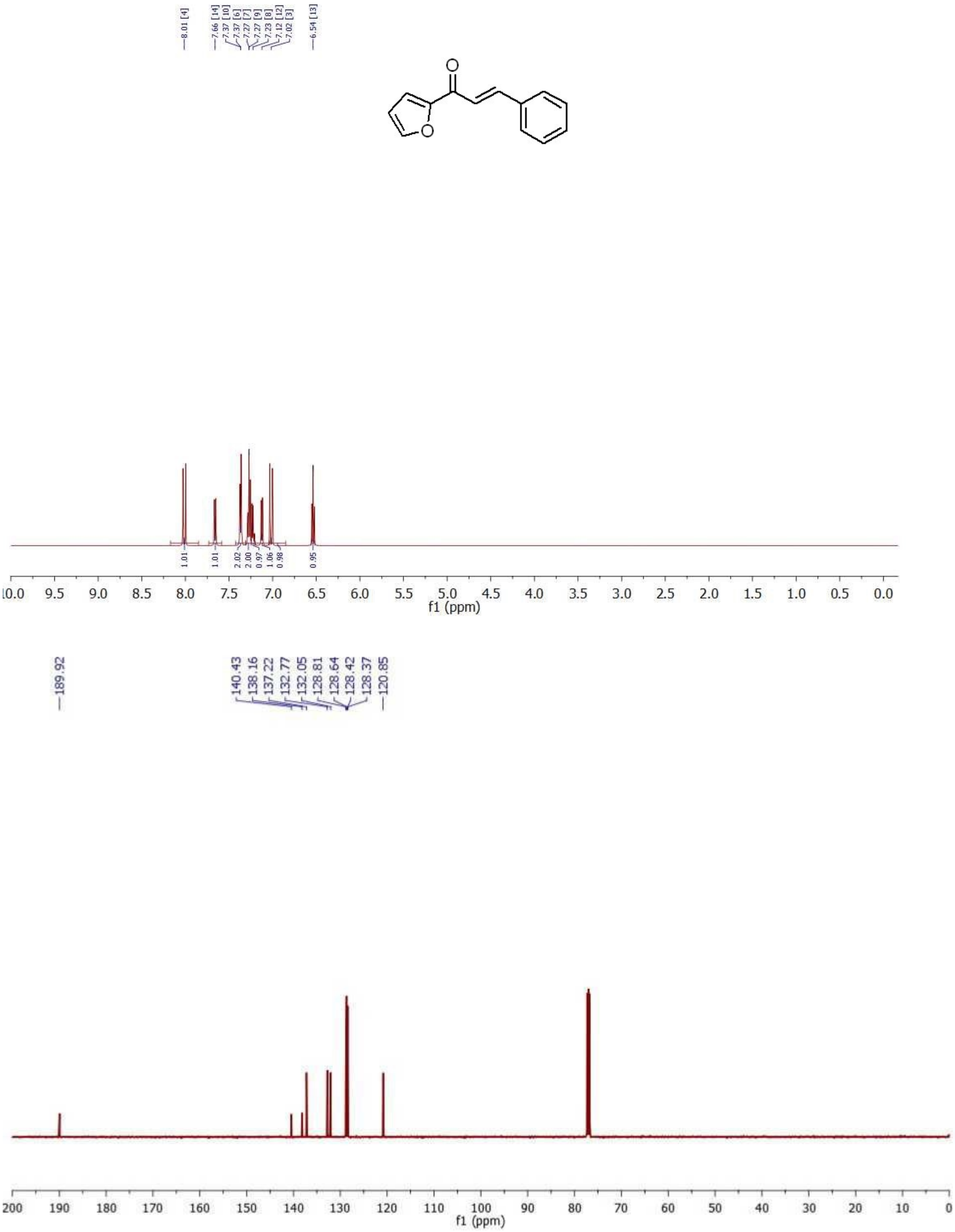

Fig.S8 ^{1}H and ^{13}C-NMR spectrum of 3h (126 MHz, $CDCl_3$)

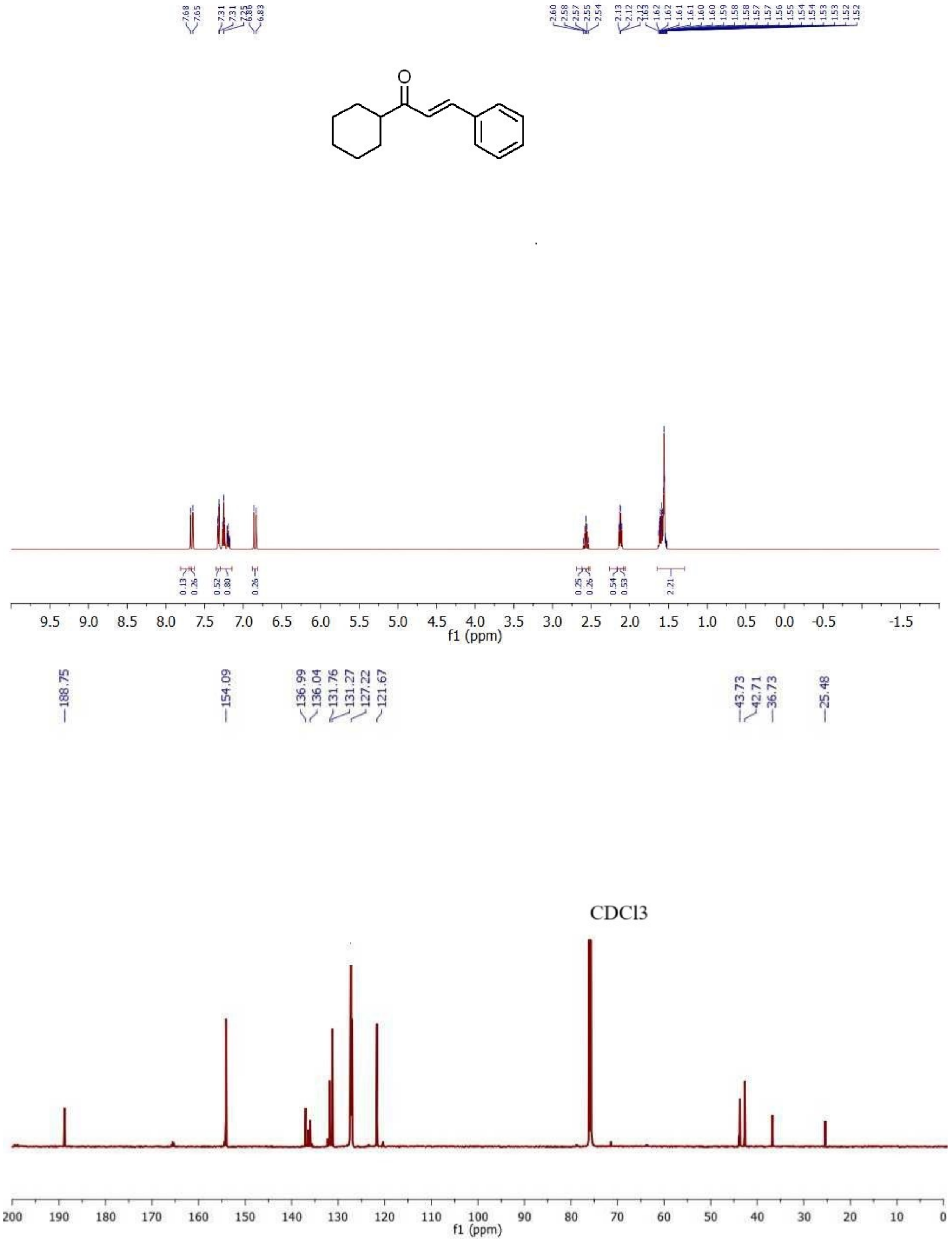

Fig.S9 ^{1}H and ^{13}C-NMR spectrum of 3i (126 MHz, $CDCl_3$)

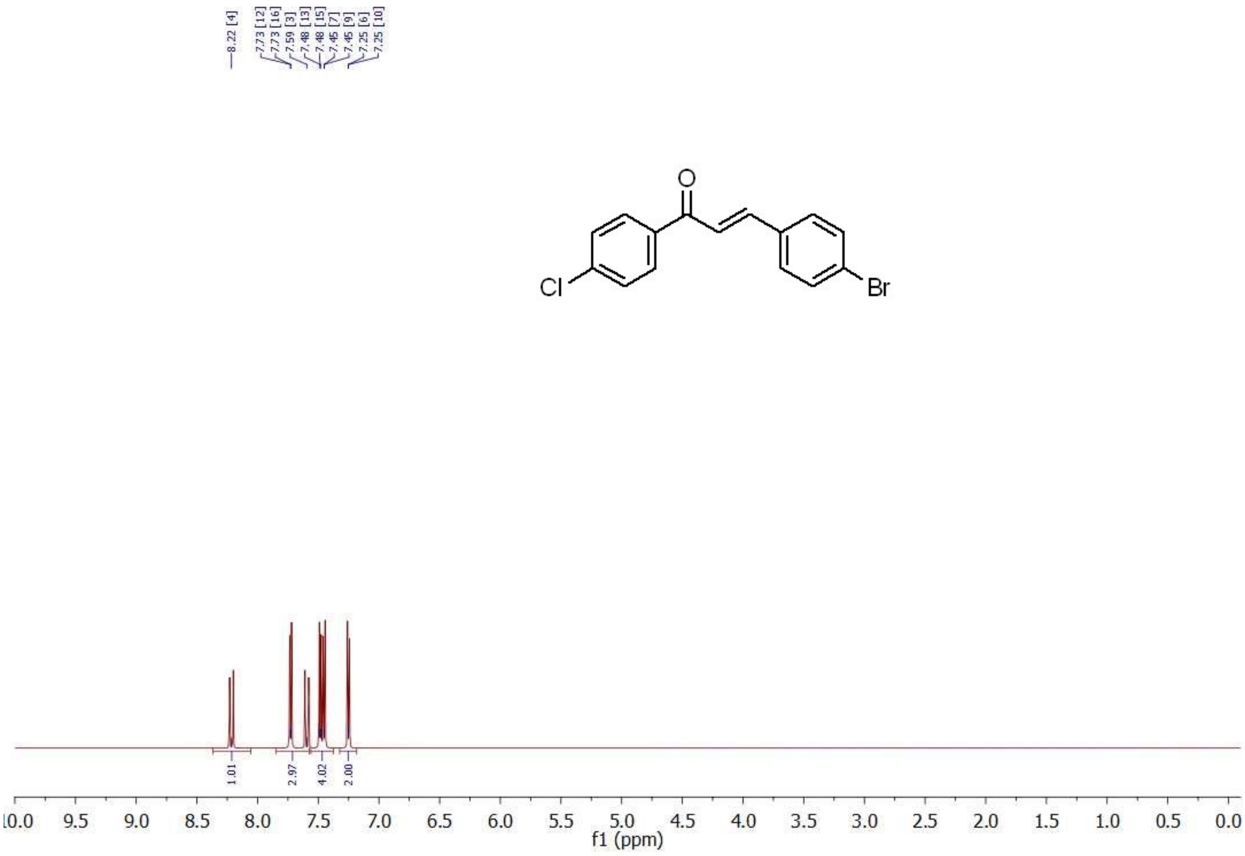

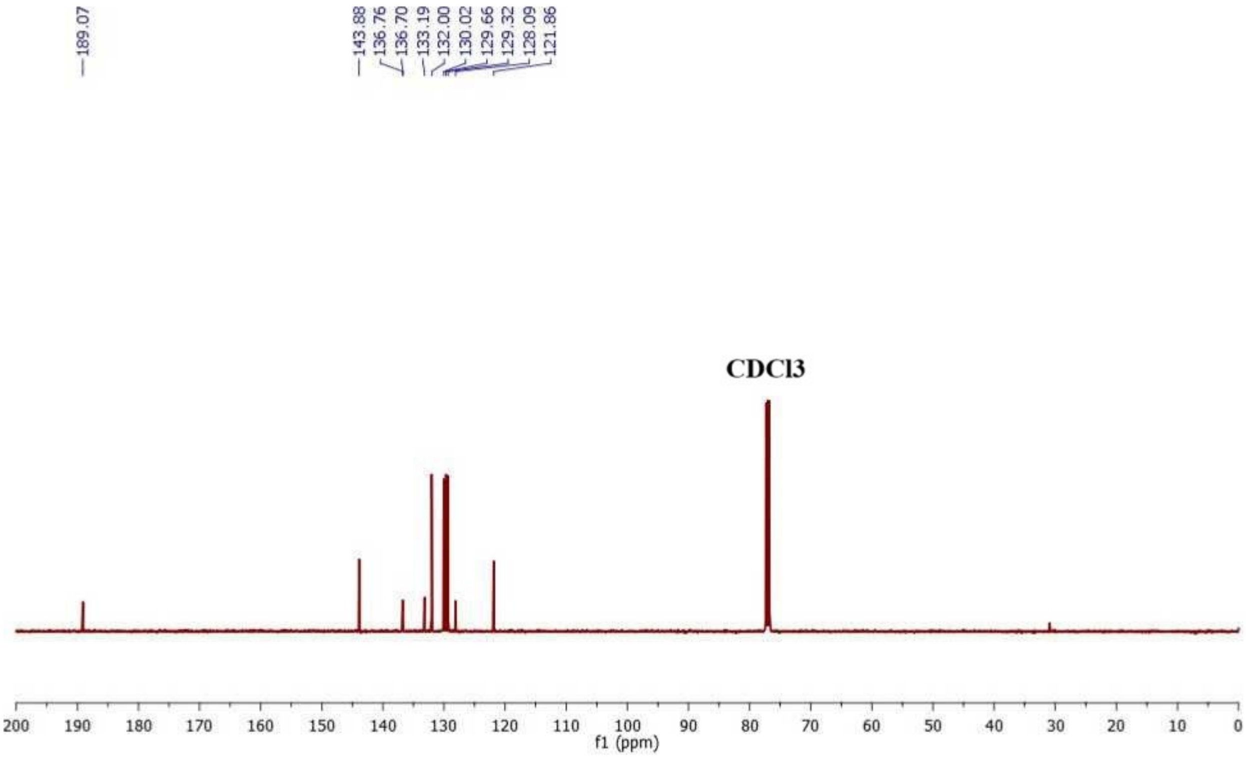

Fig.S10 ^{1}H and ^{13}C-NMR spectrum of 3jb (126 MHz, $CDCl_3$)

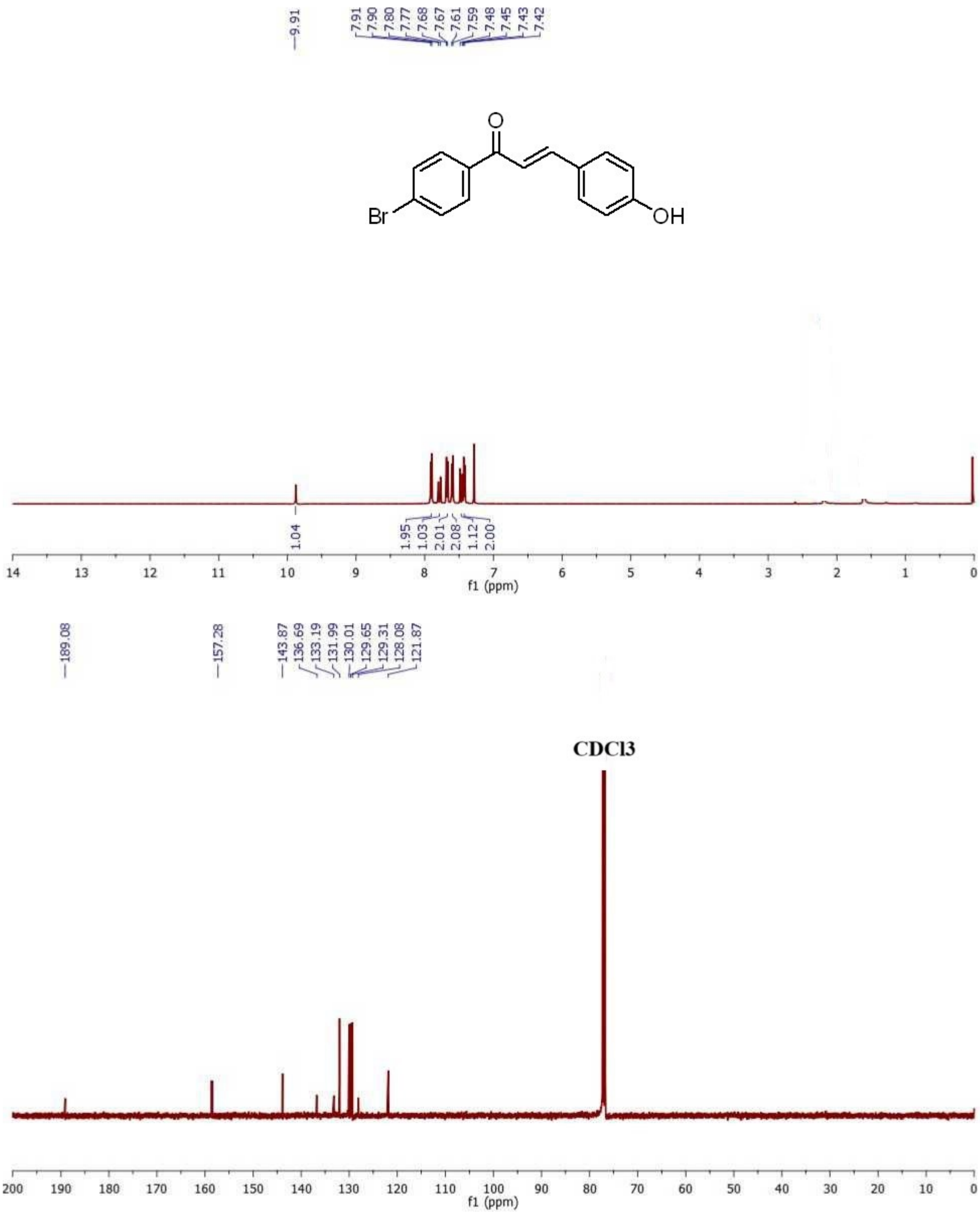

Fig.S11 ^{1}H and ^{13}C-NMR spectrum of 3kb (126 MHz, $CDCl_3$)

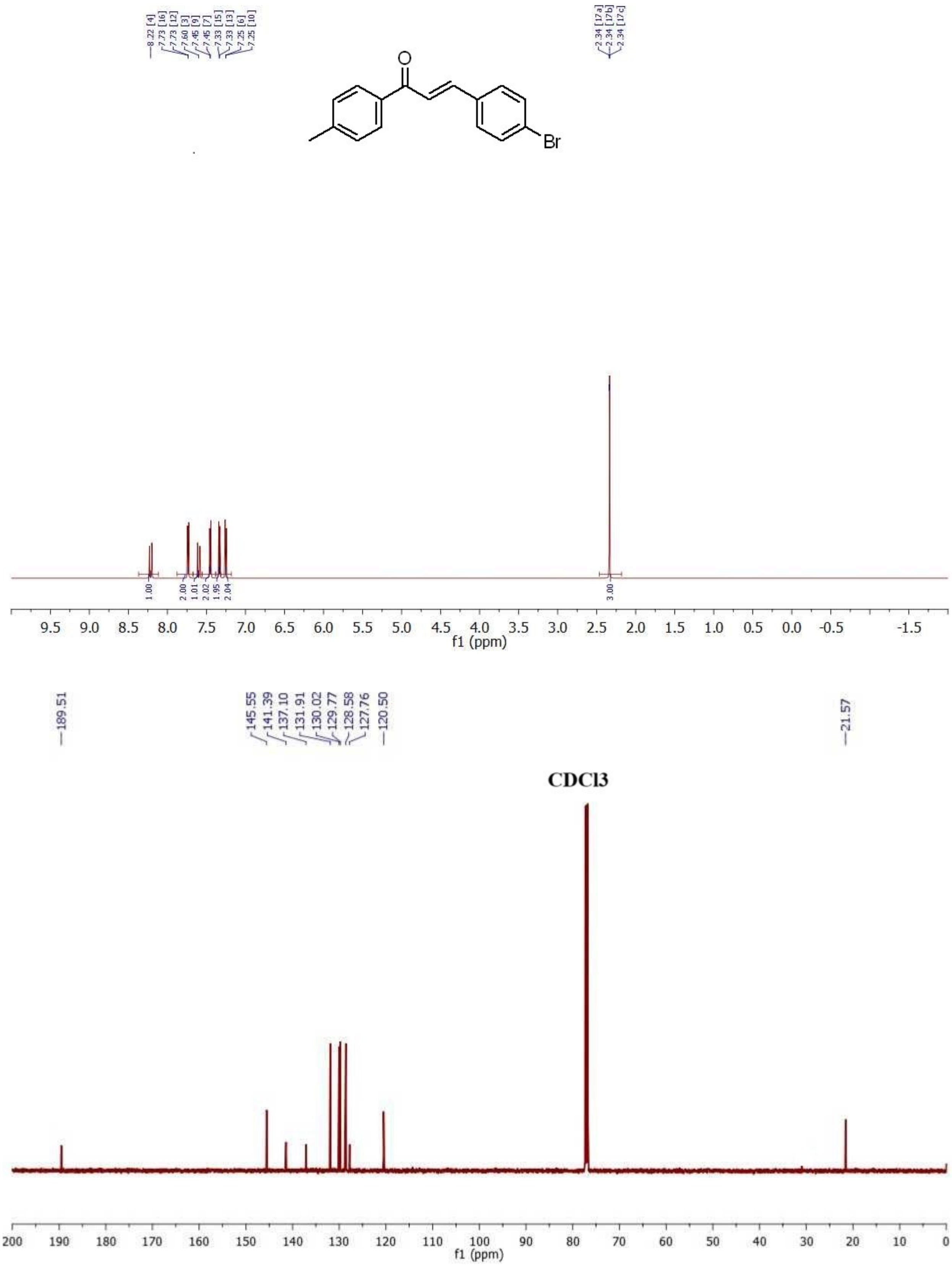

Fig.S12 ^{1}H and ^{13}C-NMR spectrum of 3lb (126 MHz, $CDCl_3$)

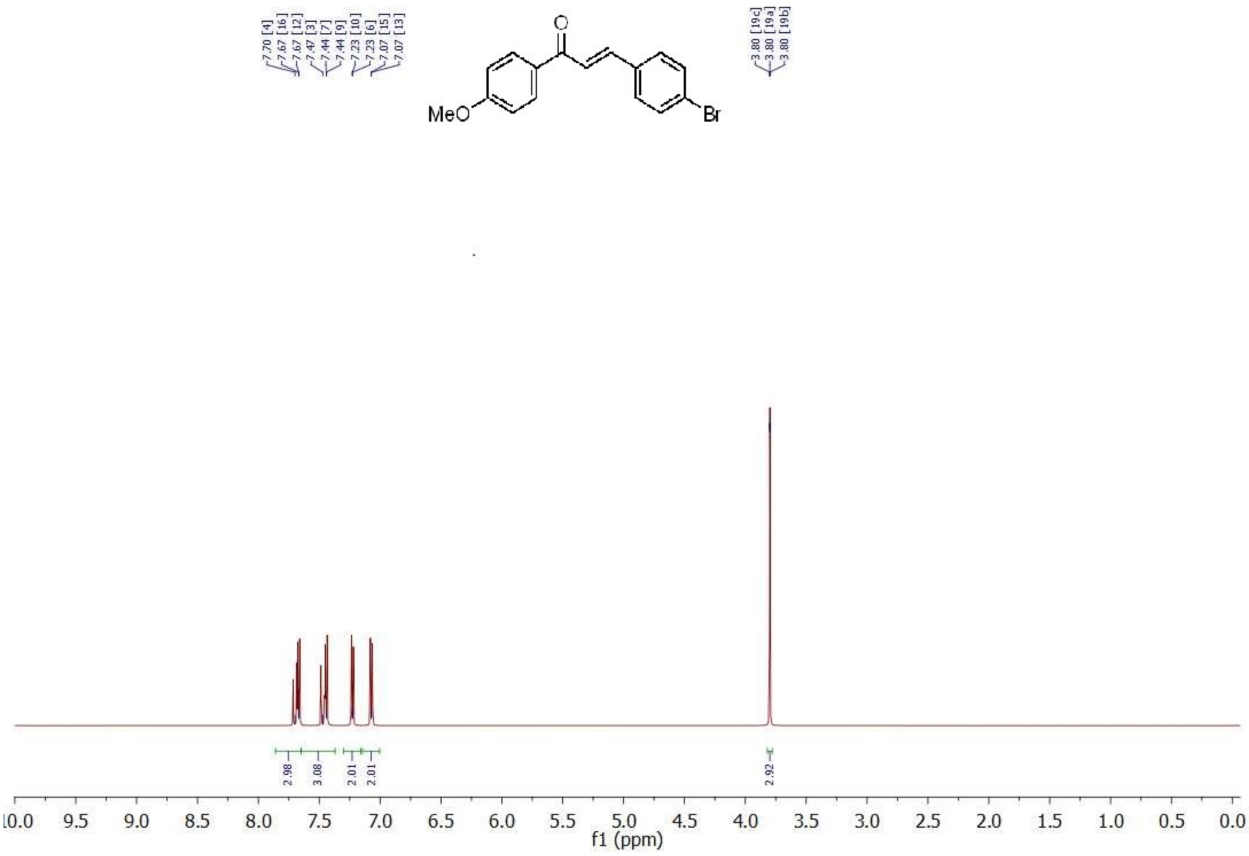

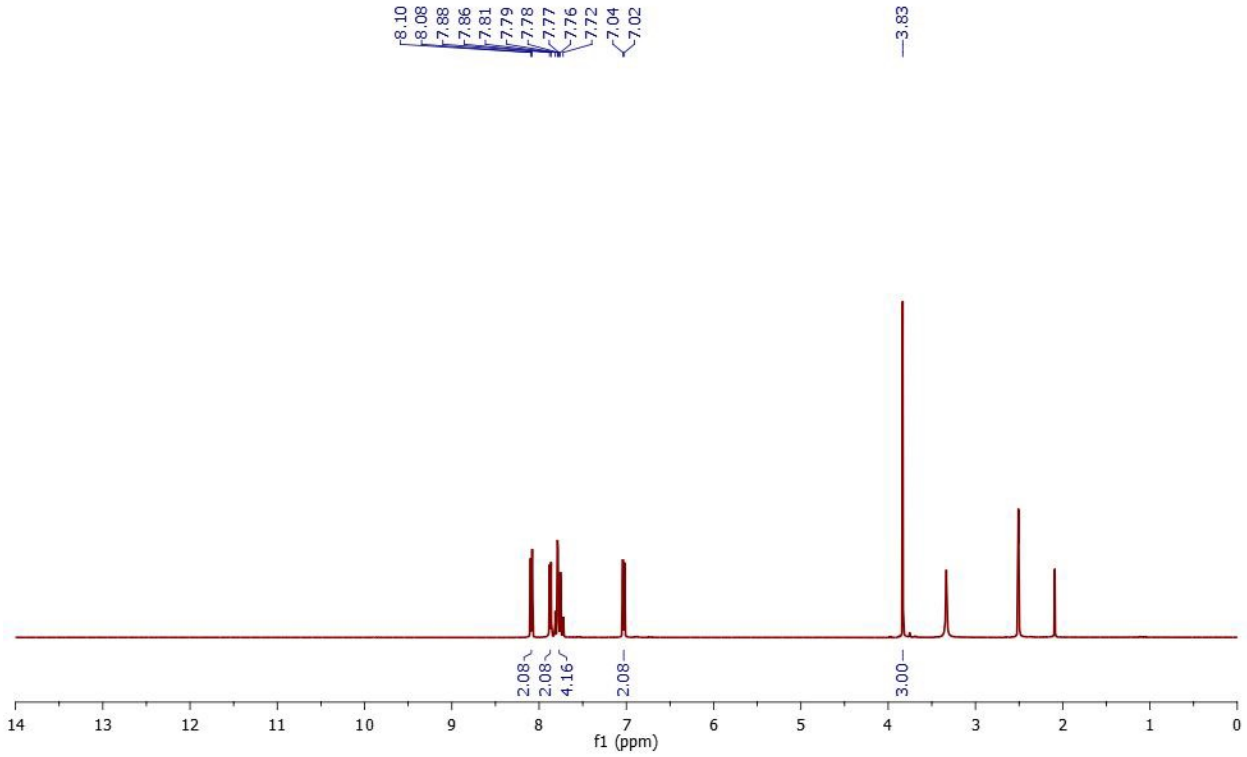

Fig.S13 ^{1}H and ^{13}C-NMR spectrum of 3mb (126 MHz, $CDCl_3$)

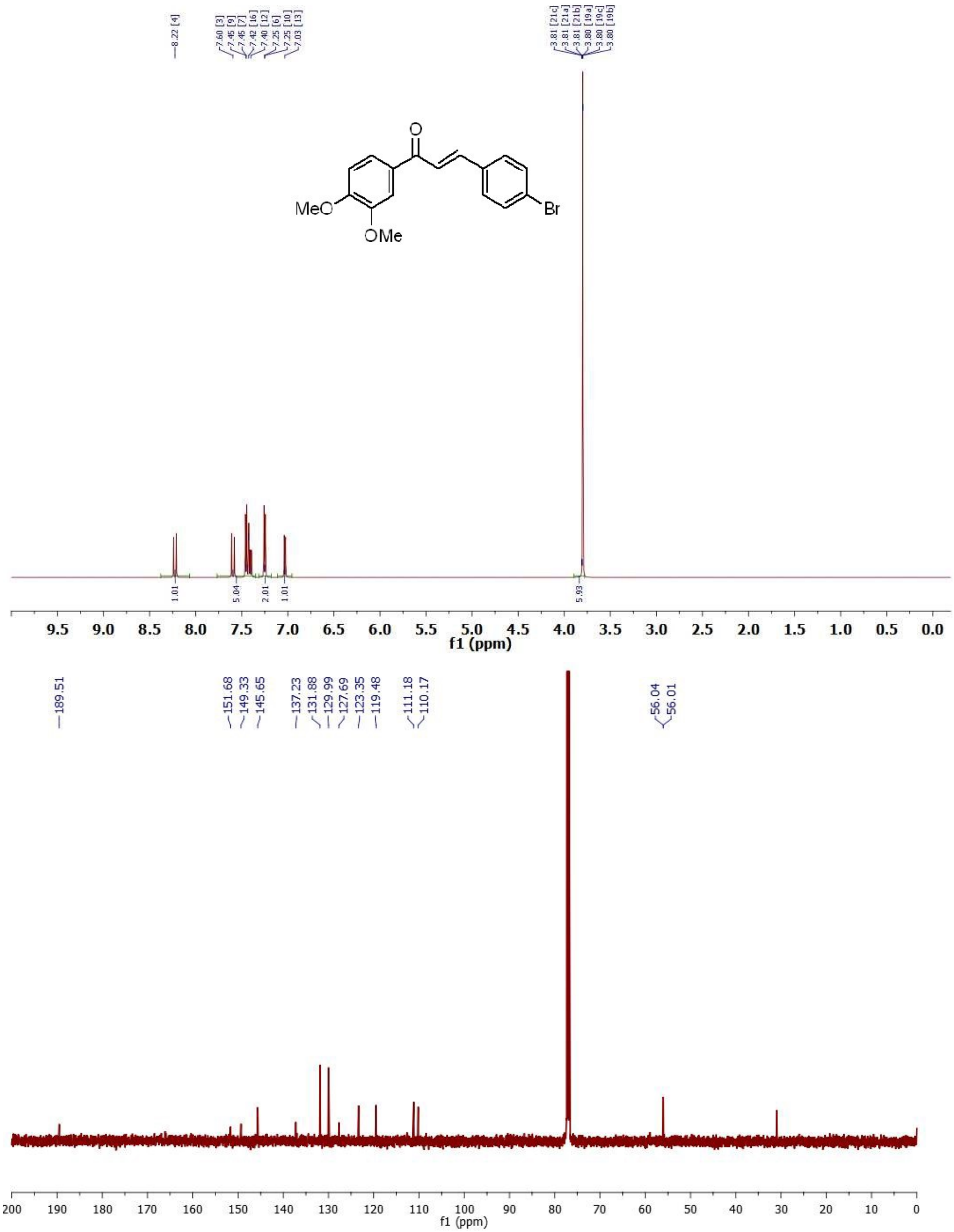

Fig.S14 ^{1}H and ^{13}C-NMR spectrum of 3nb (126 MHz, $CDCl_3$)

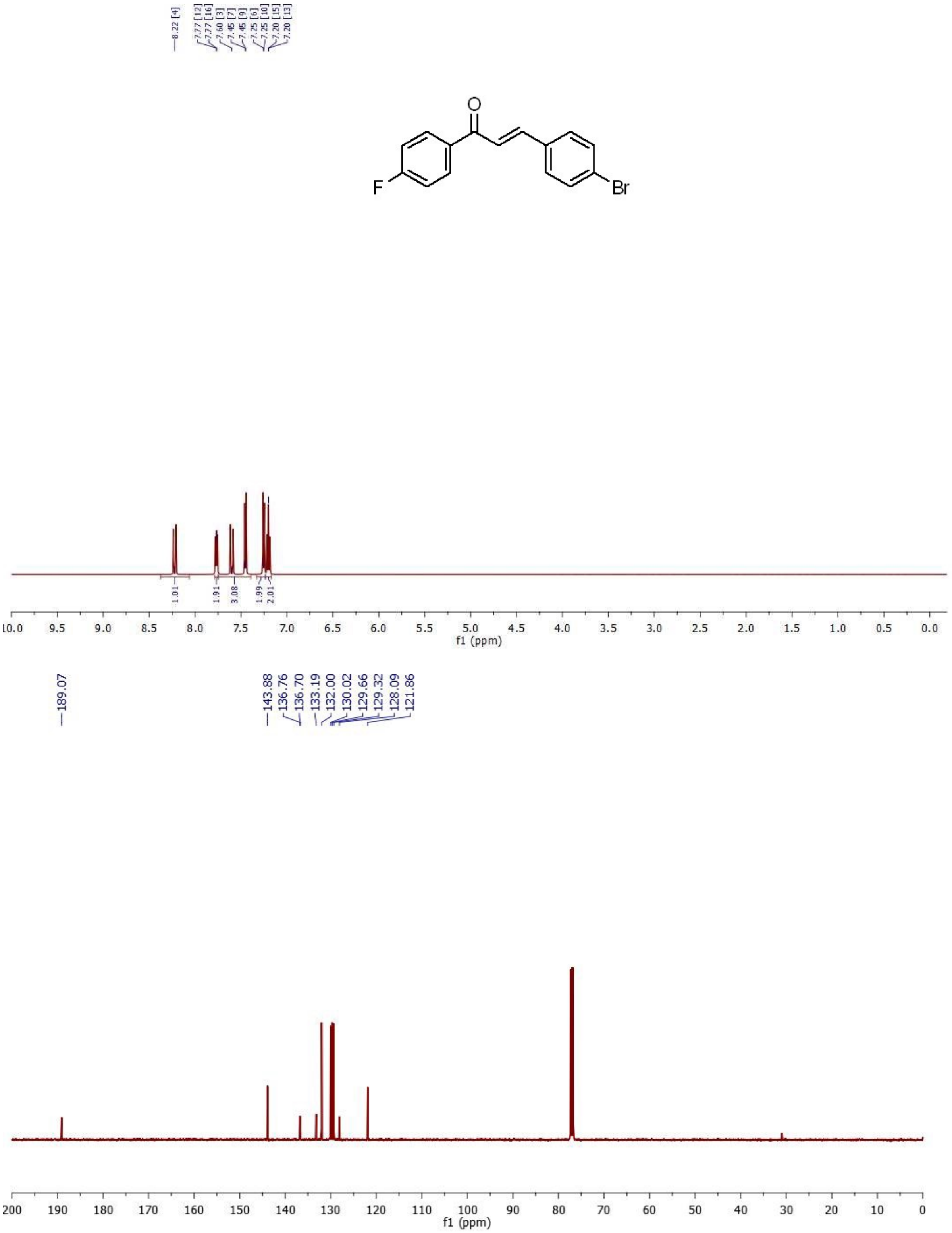

Fig.S15 ^{1}H and ^{13}C-NMR spectrum of 3ob (126 MHz, $CDCl_3$)

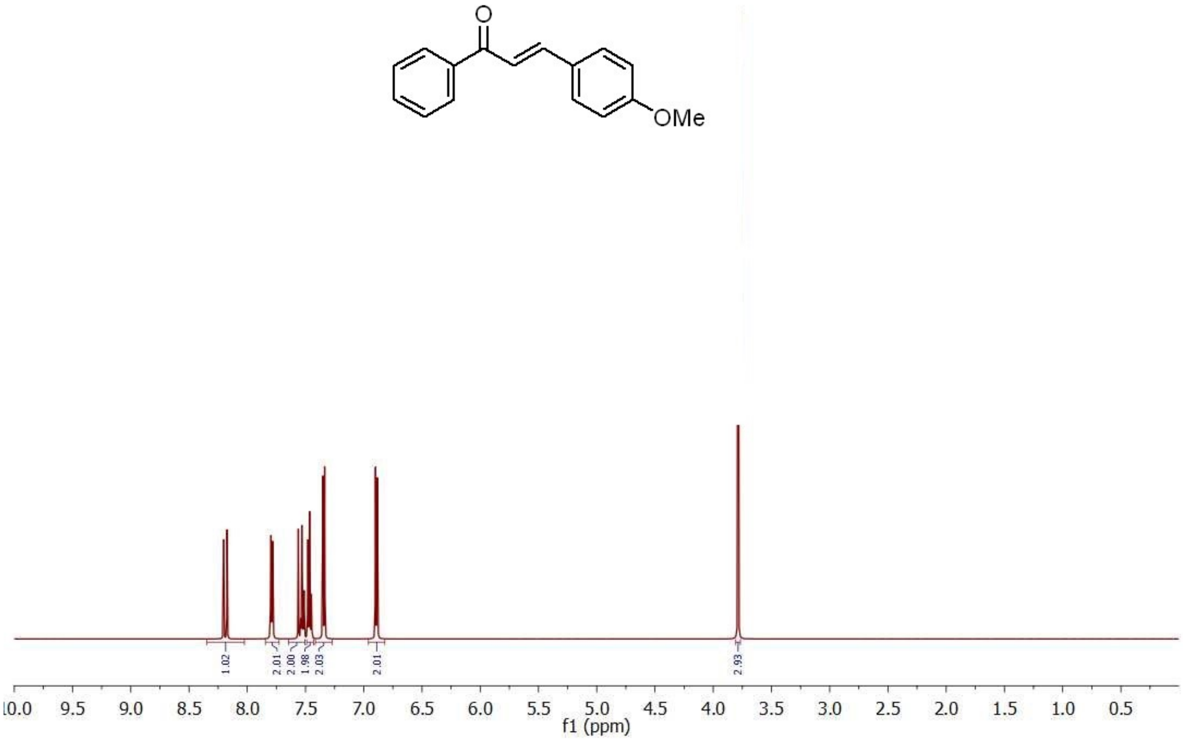

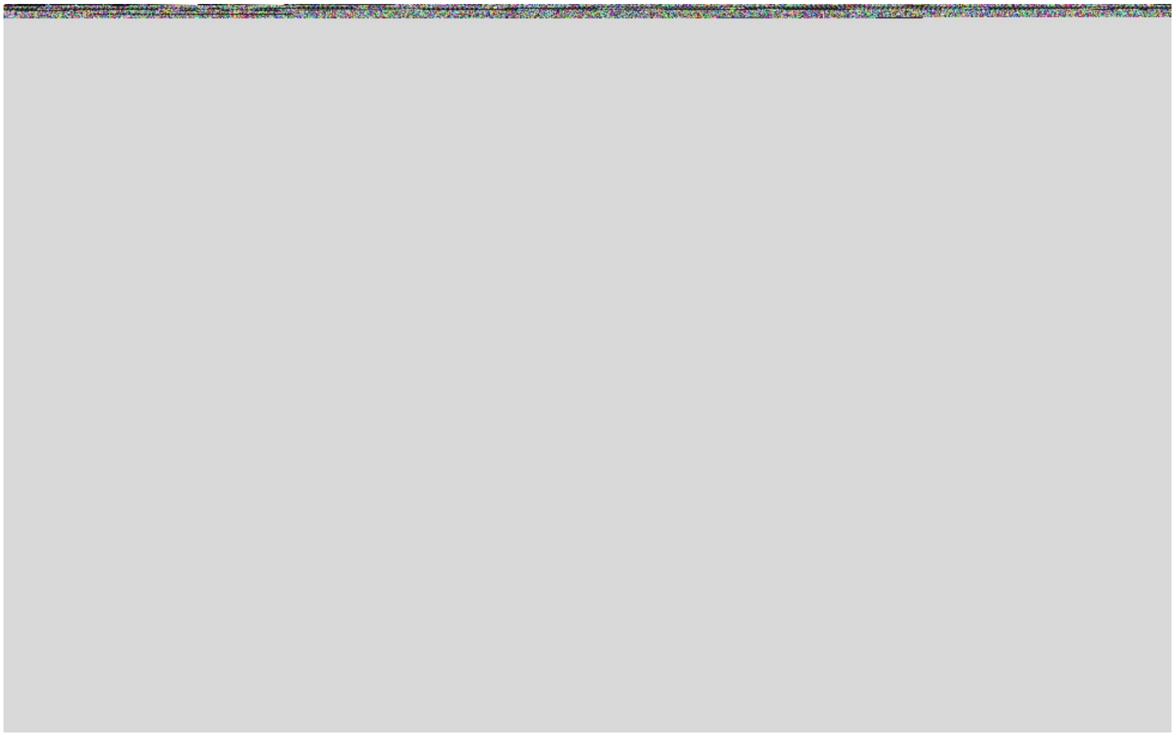

Fig.S16 ^{1}H and ^{13}C-NMR spectrum of 3bc (126 MHz, $CDCl_3$)

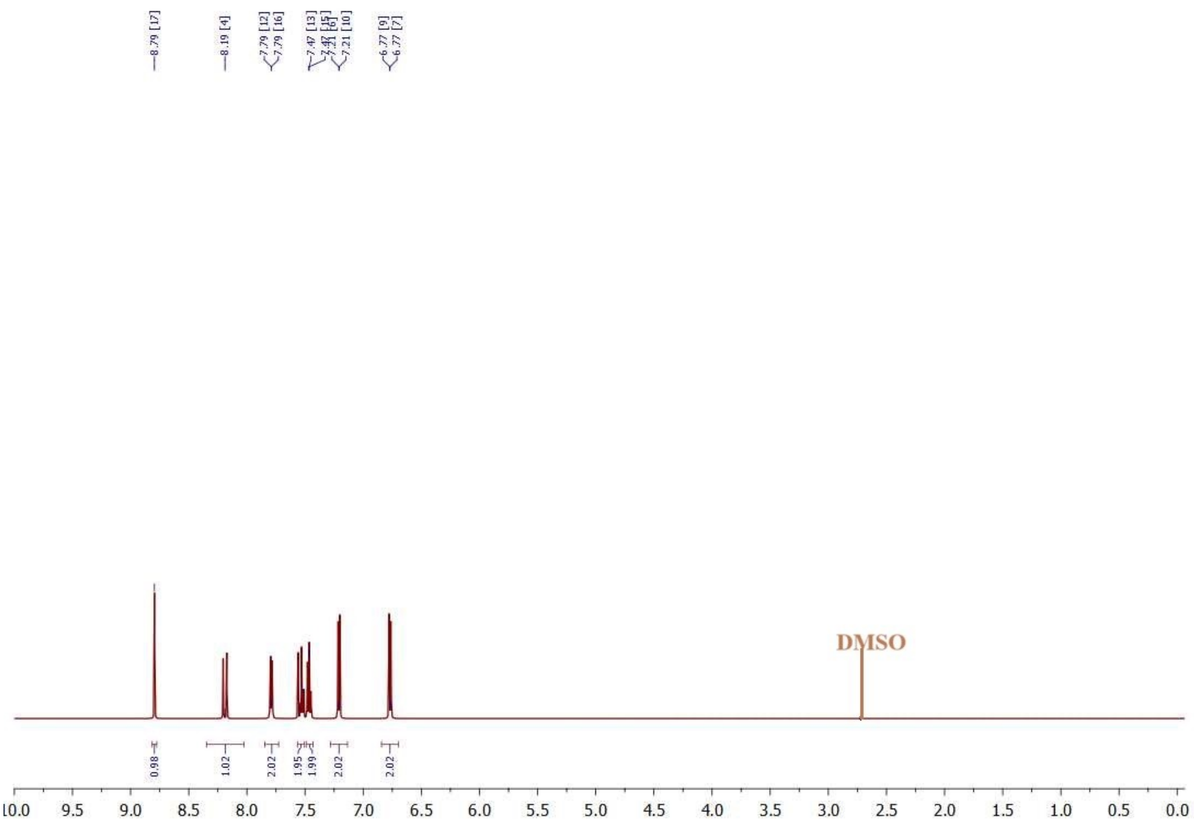

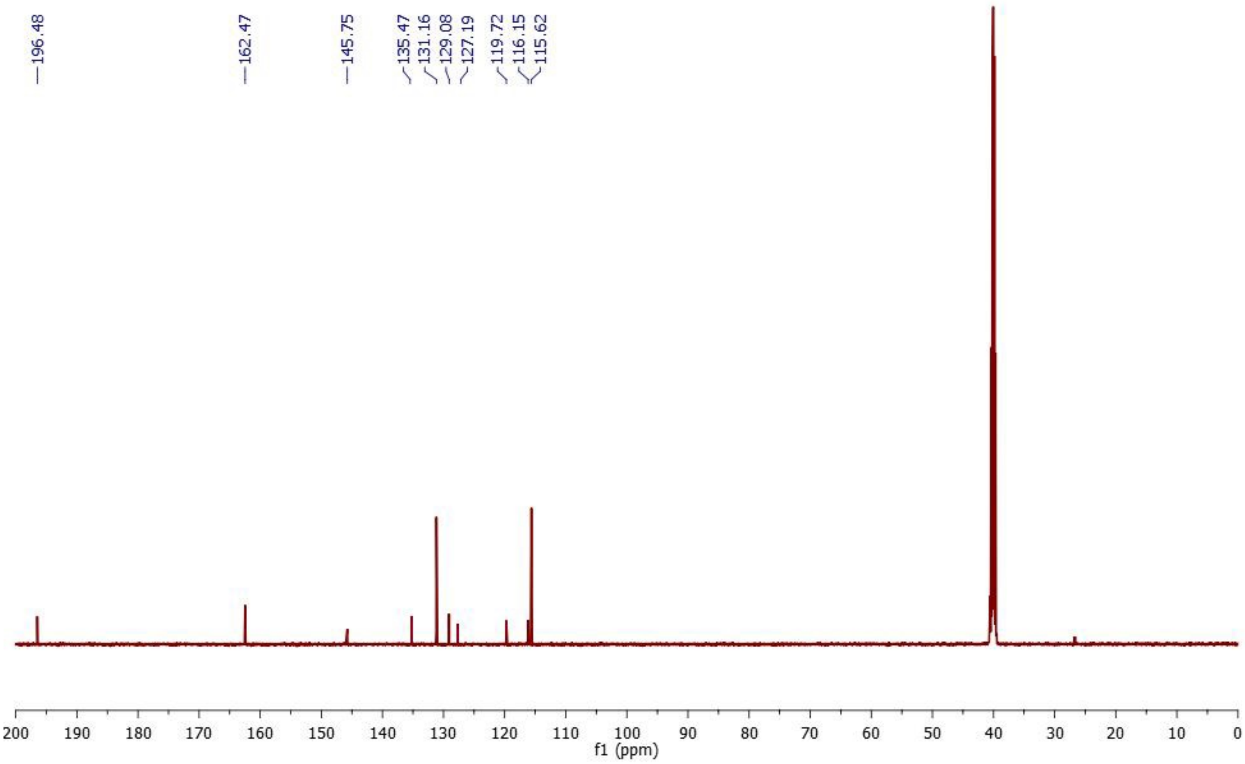

Fig.S17 **^{1}H and ^{13}C-NMR spectrum of 3bd (126 MHz, $CDCl_3$)**

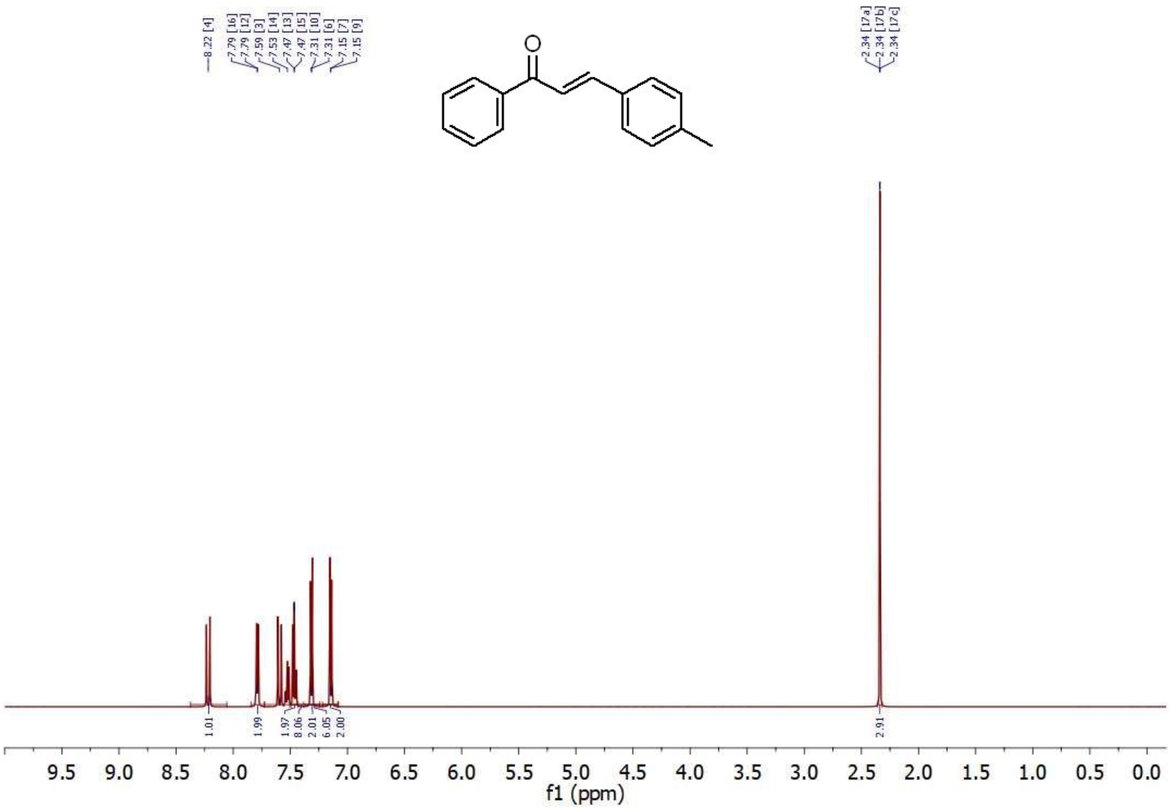

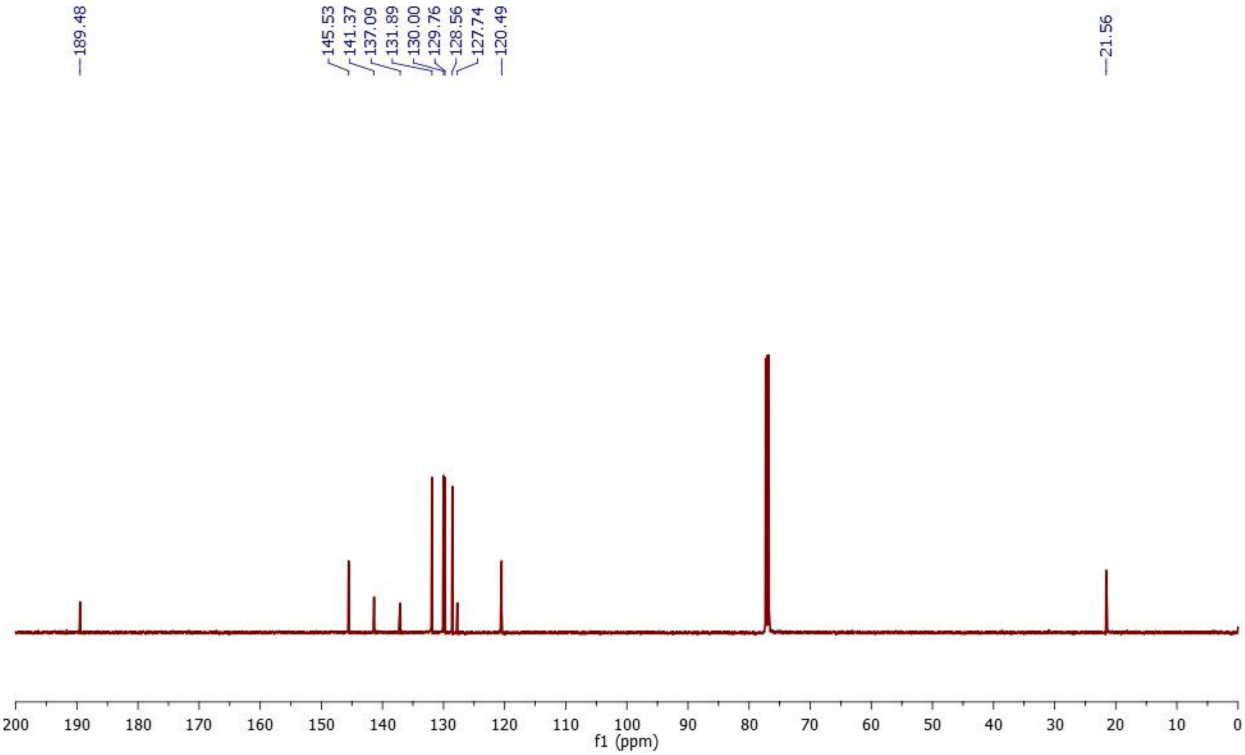

Fig.S18 ^{1}H and ^{13}C-NMR spectrum of 3be (126 MHz, $CDCl_3$)

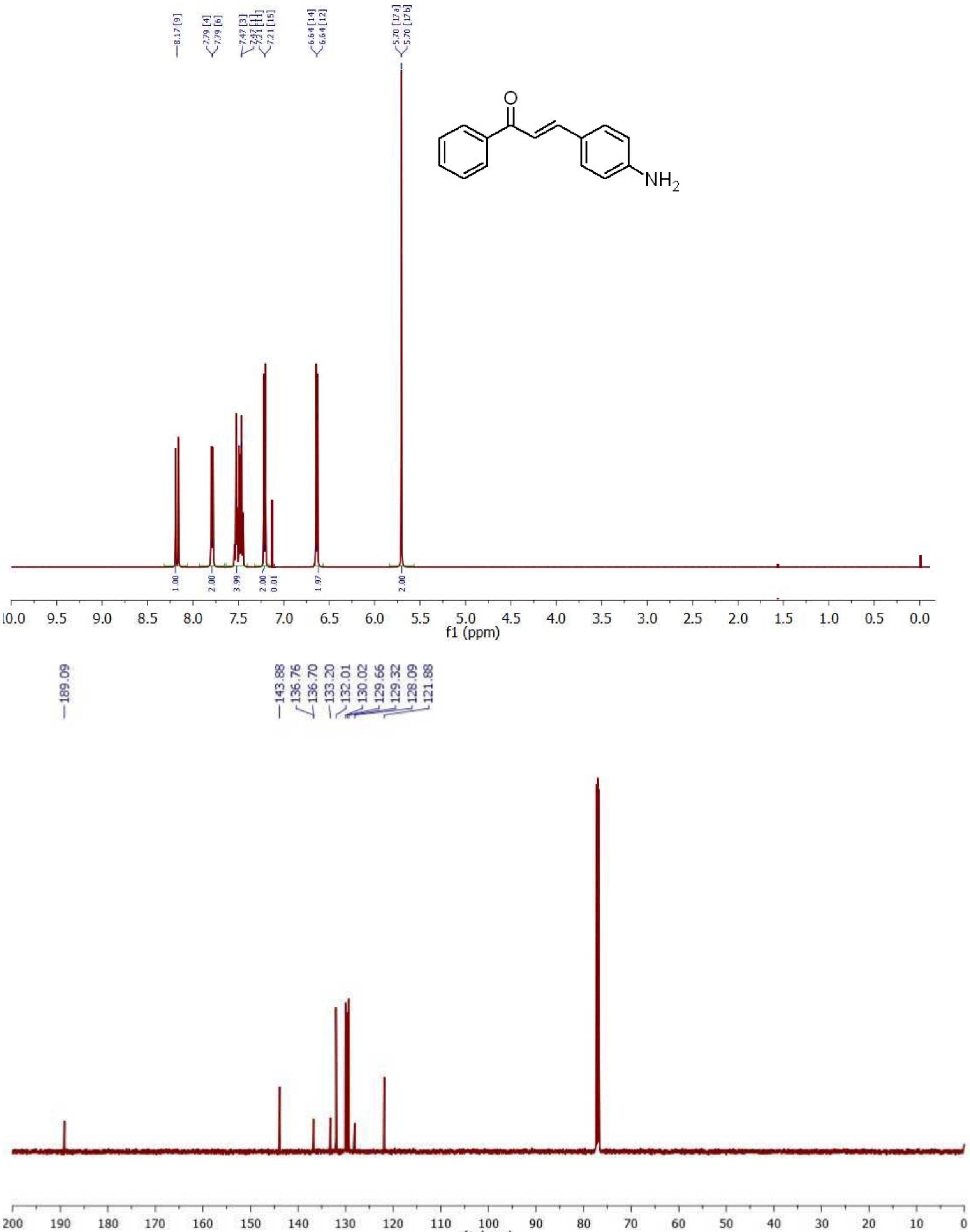

Fig.S19 ^{1}H and ^{13}C-NMR spectrum of 3bf (126 MHz, $CDCl_3$)

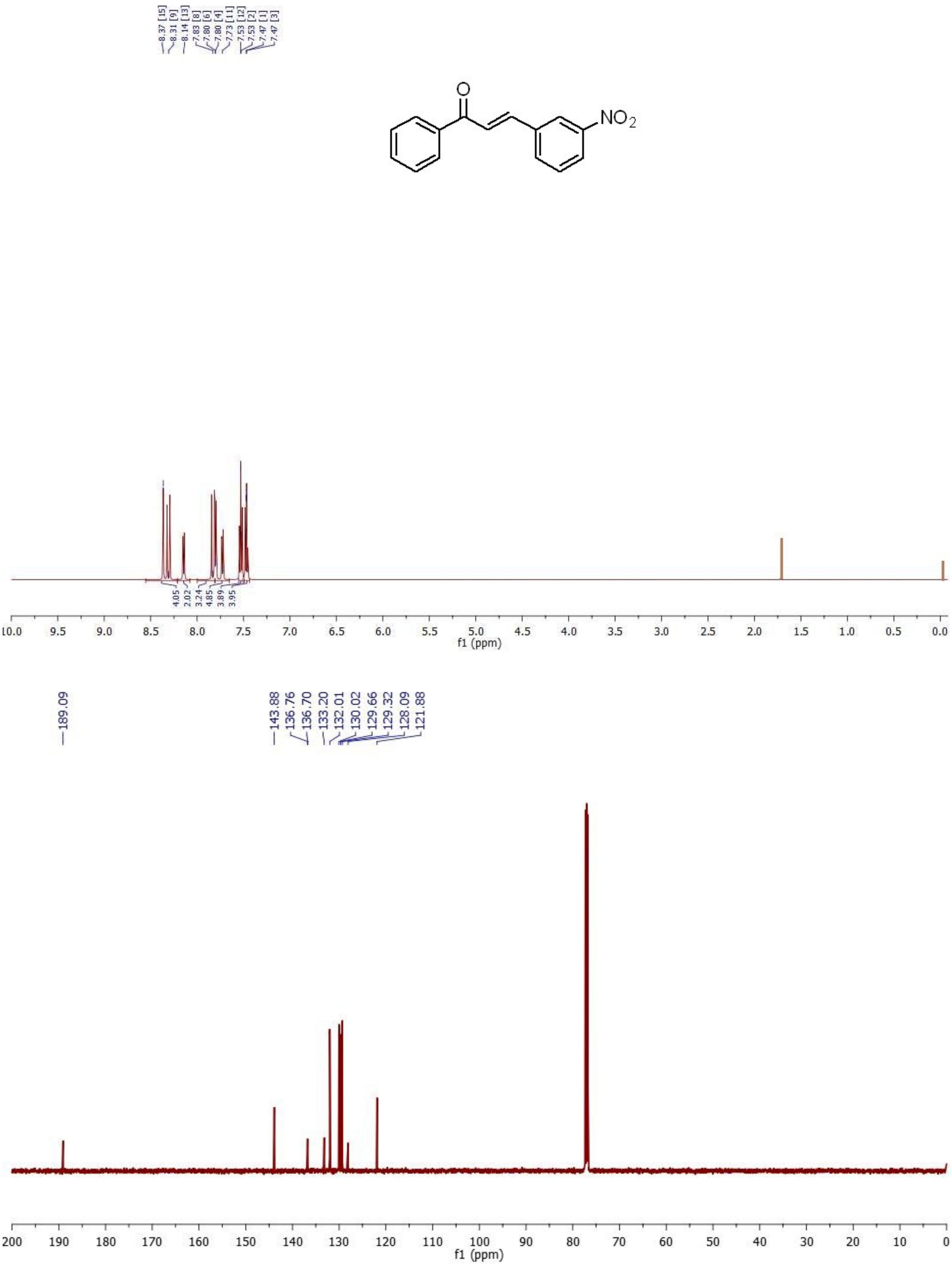

Fig.S20 ^{1}H and ^{13}C-NMR spectrum of 3bf (126 MHz, $CDCl_3$)

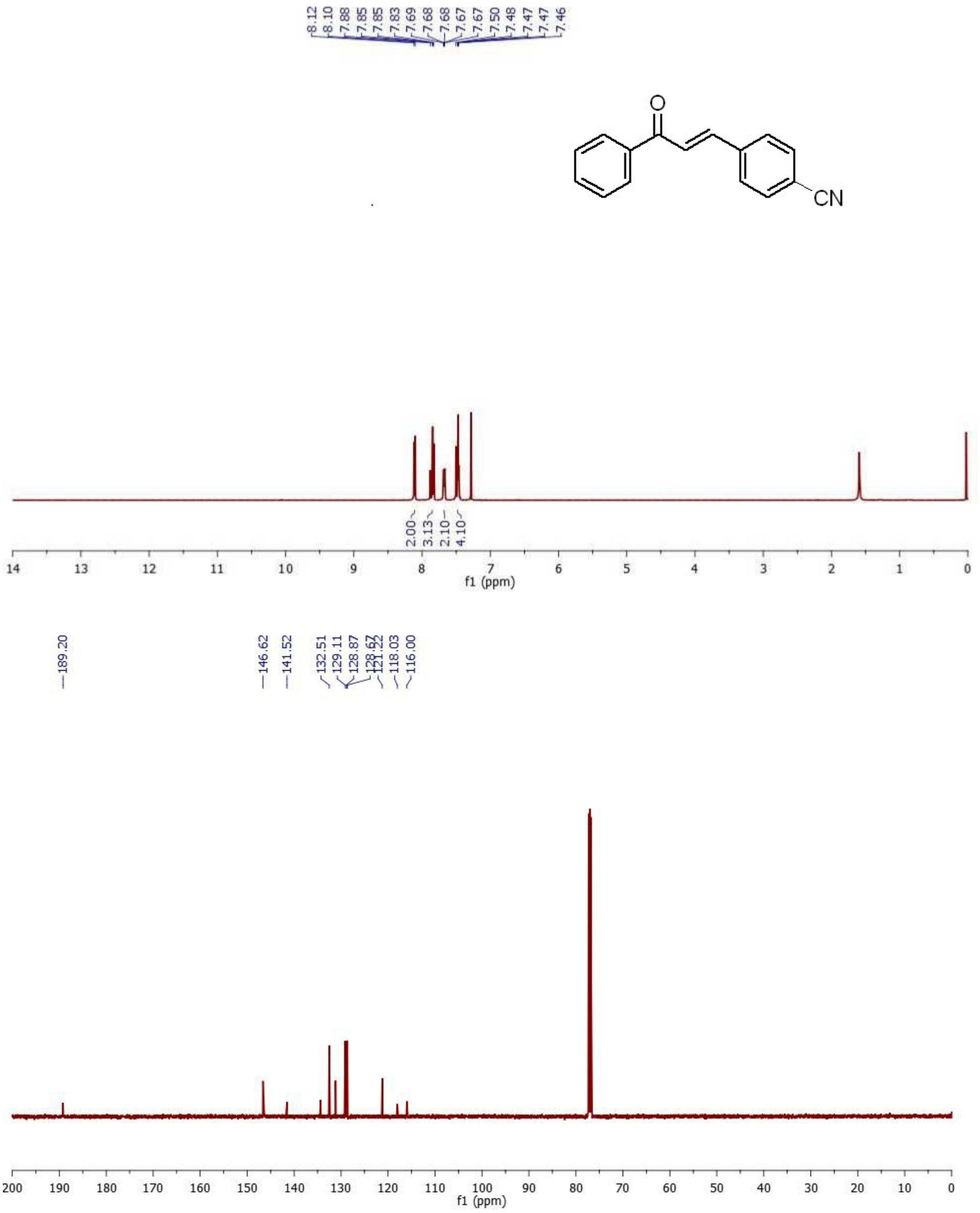

Fig.S21 ^{1}H and ^{13}C-NMR spectrum of 3bg (126 MHz, $CDCl_3$)

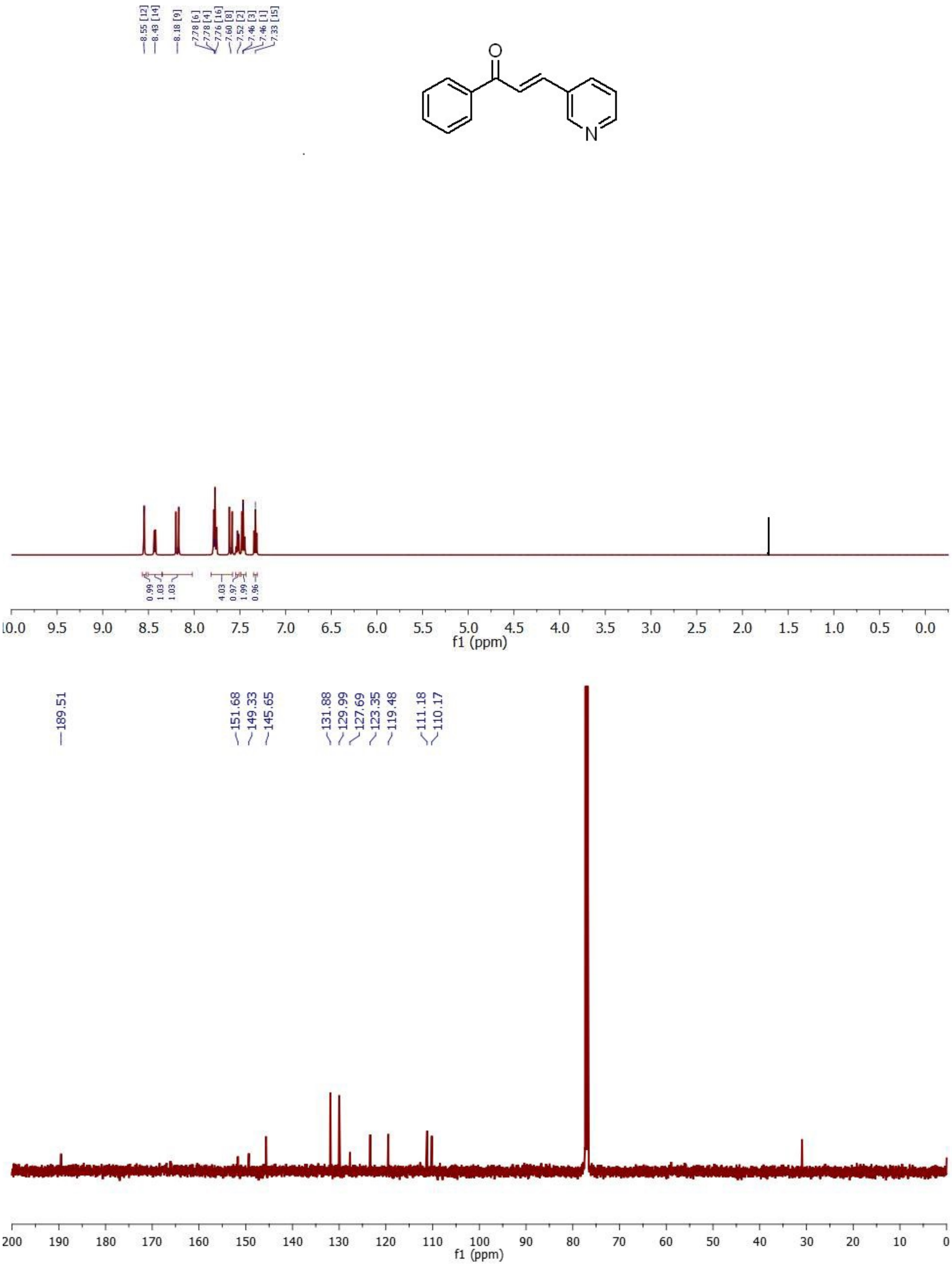

Fig.S22 **^{1}H and ^{13}C-NMR spectrum of 3bh (126 MHz, $CDCl_3$)**

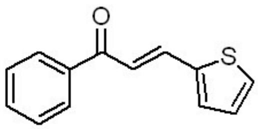

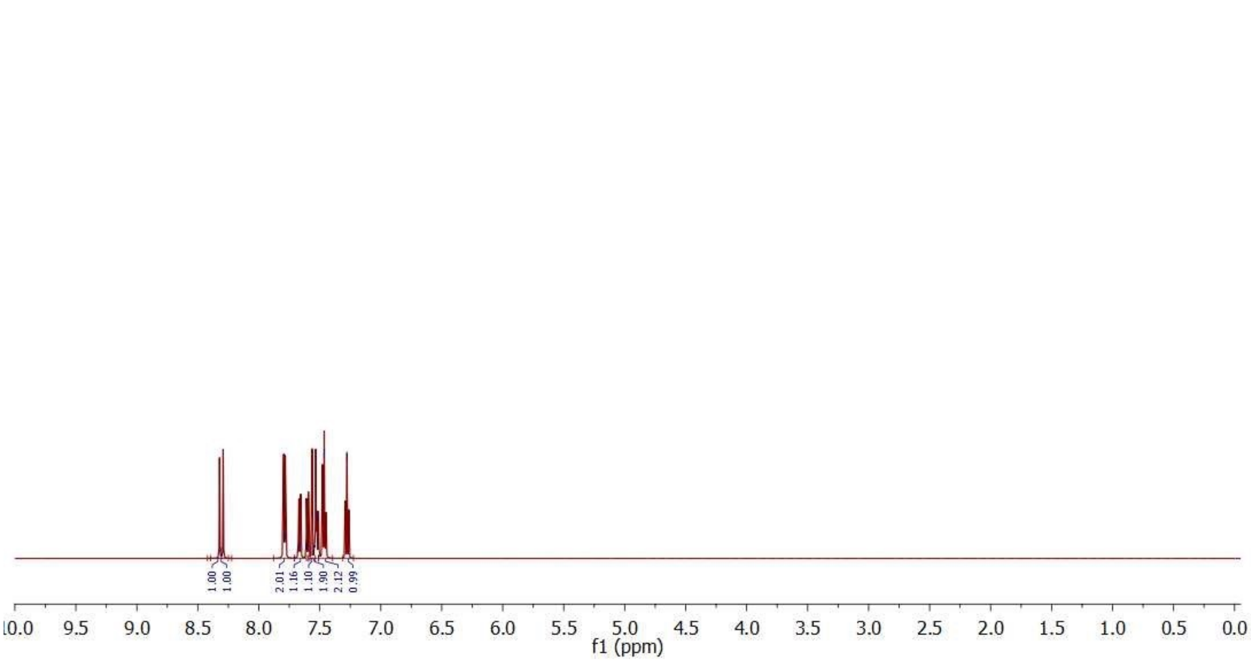

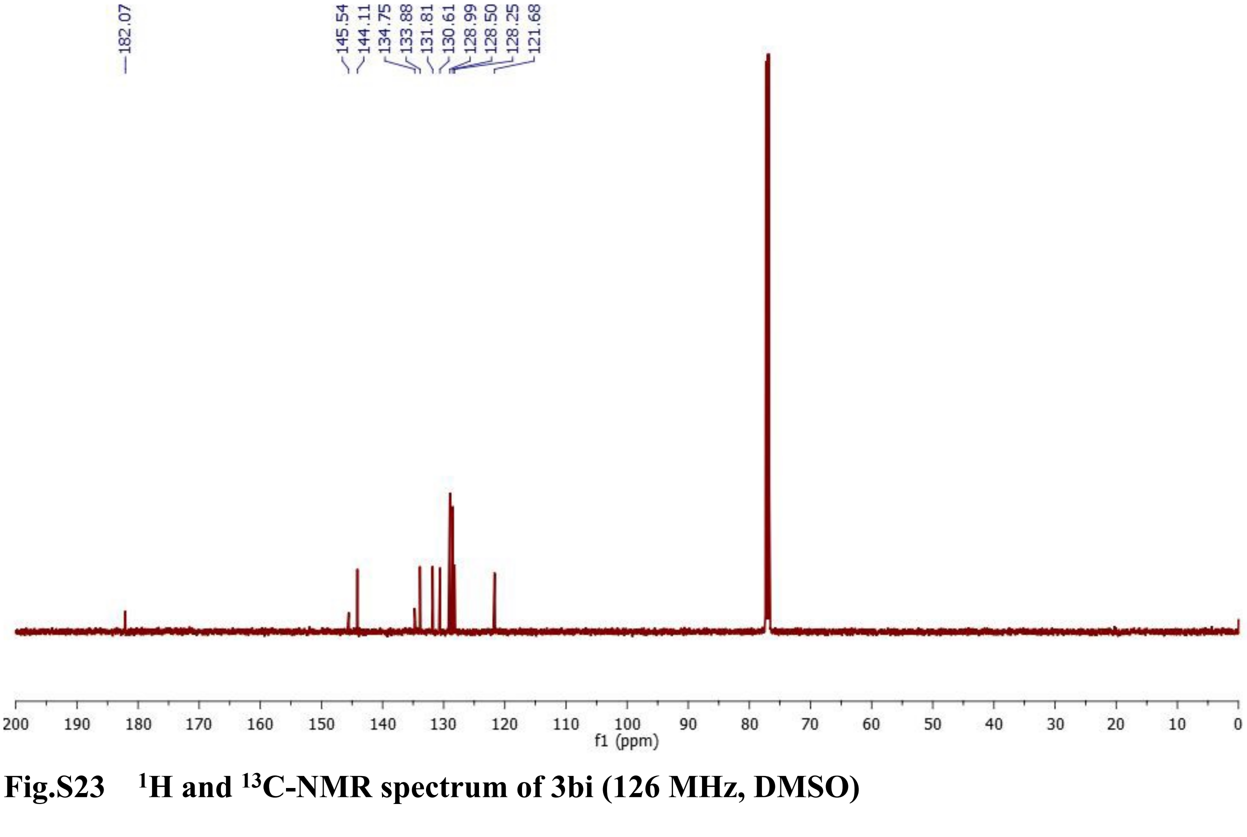

Fig.S23 ^{1}H and ^{13}C-NMR spectrum of 3bi (126 MHz, DMSO)

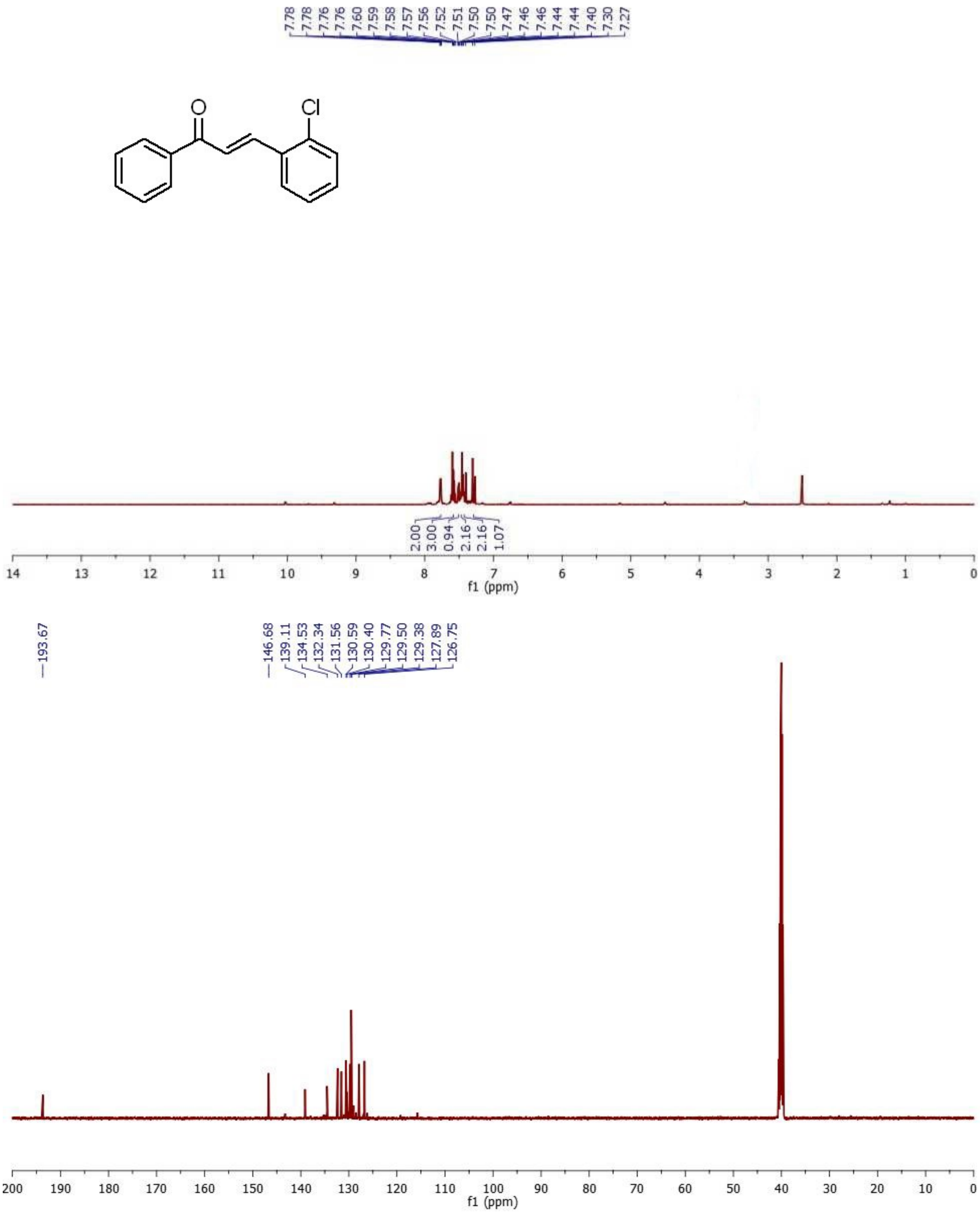

Fig.S24 ^{1}H and ^{13}C-NMR spectrum of 3bg (126 MHz, DMSO)

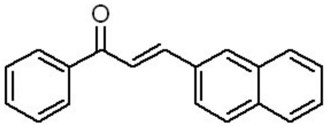

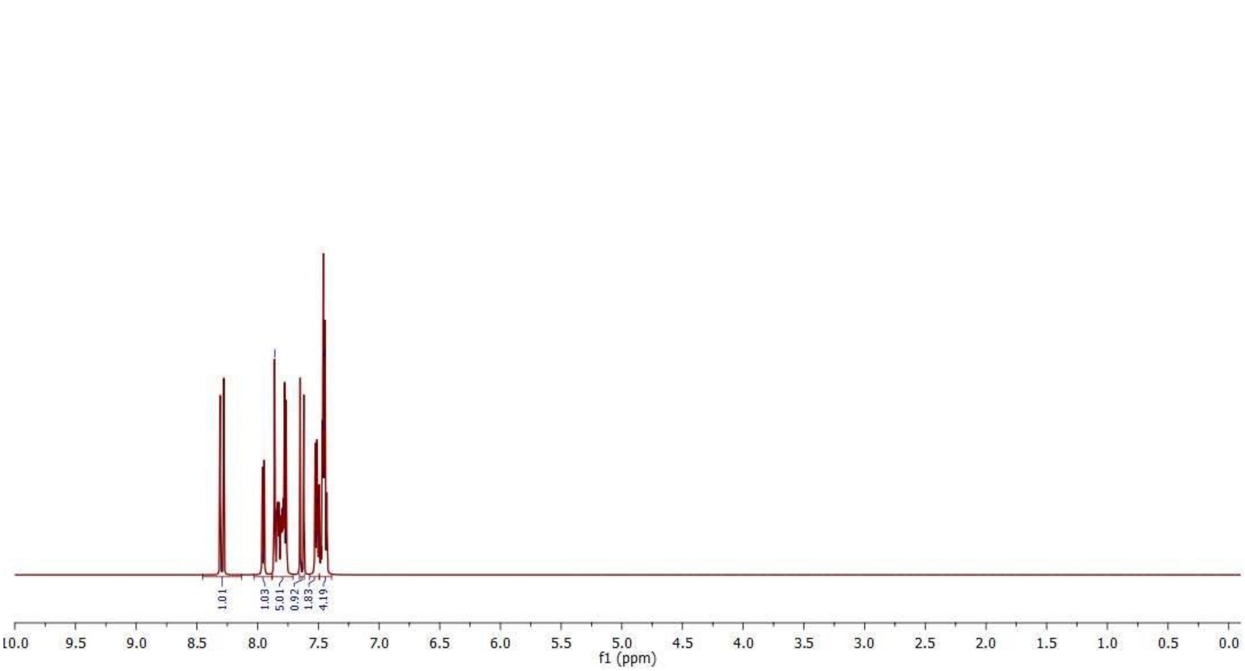

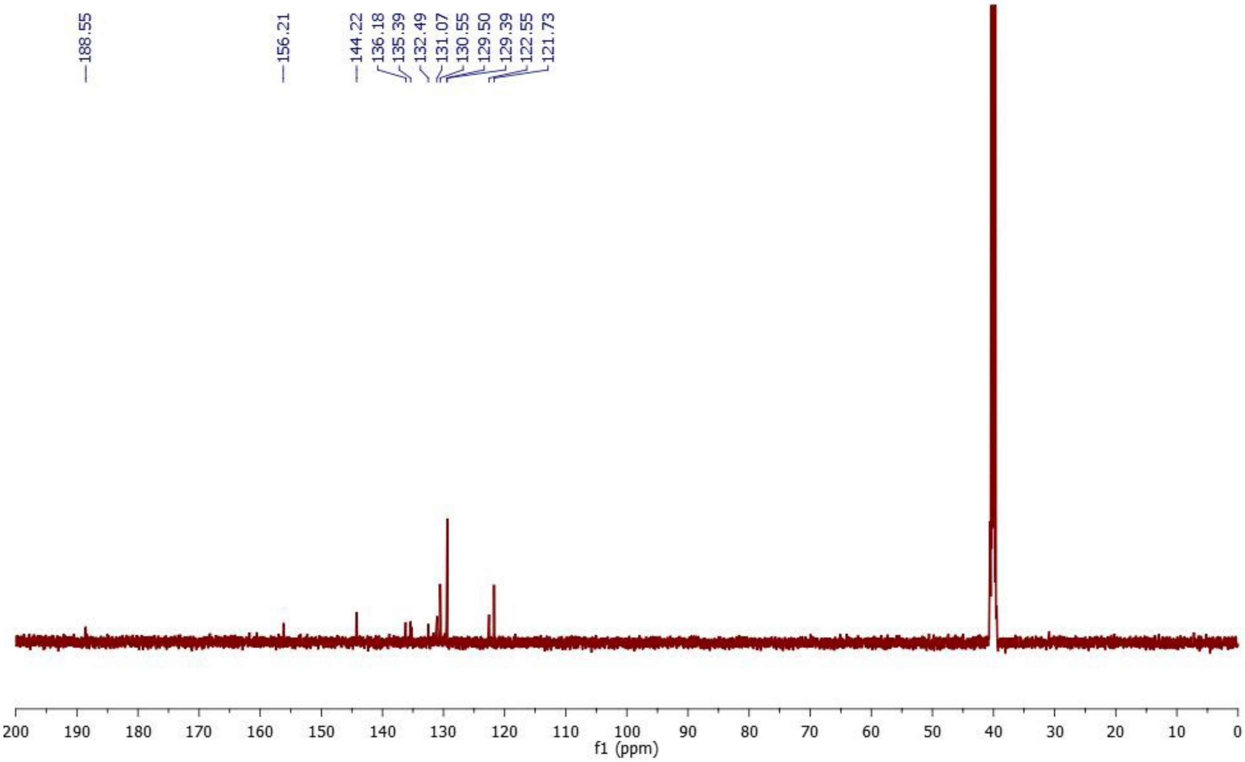

Fig.S25 ^{1}H and ^{13}C-NMR spectrum of 3bk (126 MHz, DMSO)

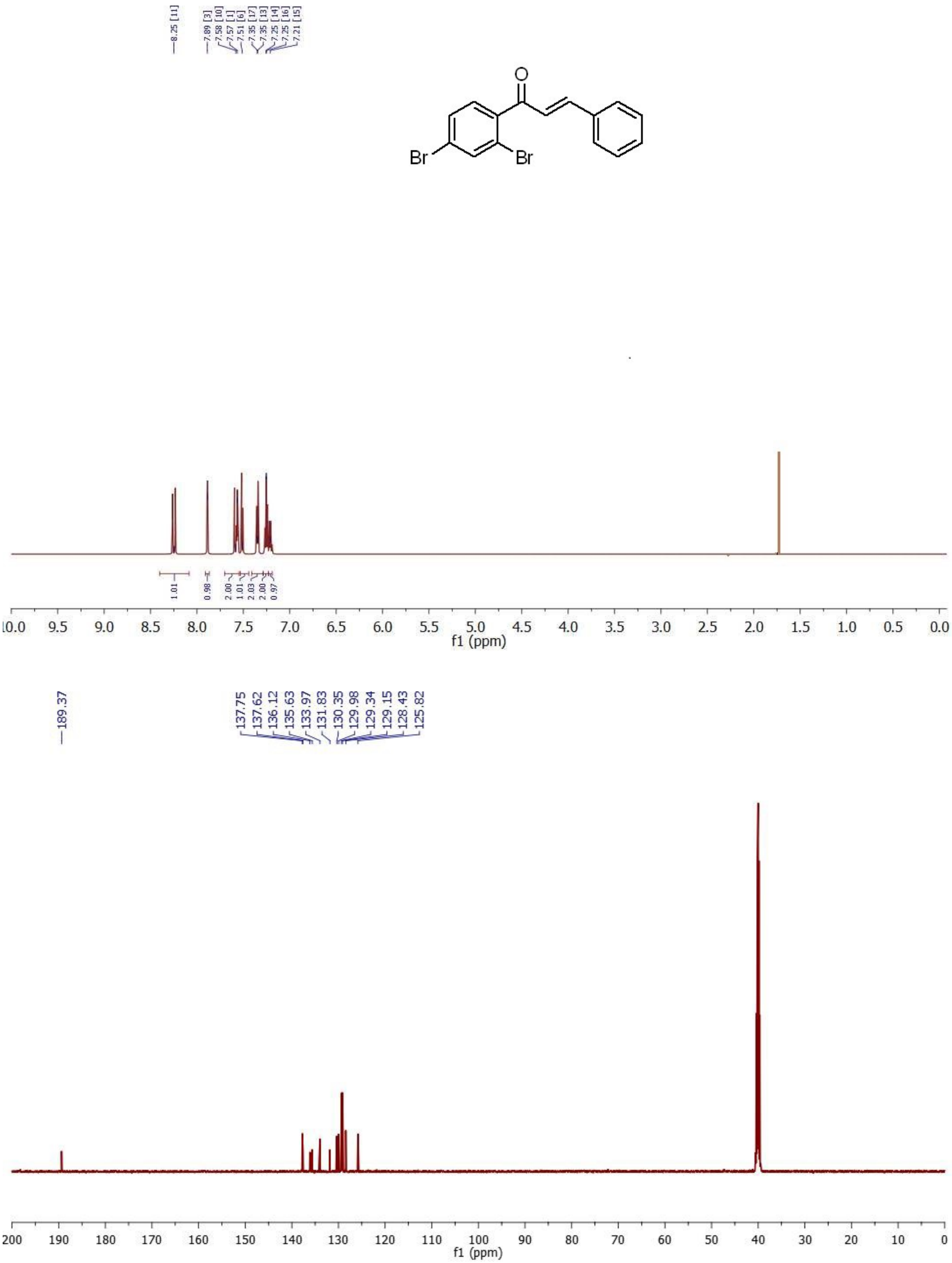

Fig.S26 ^{1}H and ^{13}C-NMR spectrum of 3bh (126 MHz, DMSO)

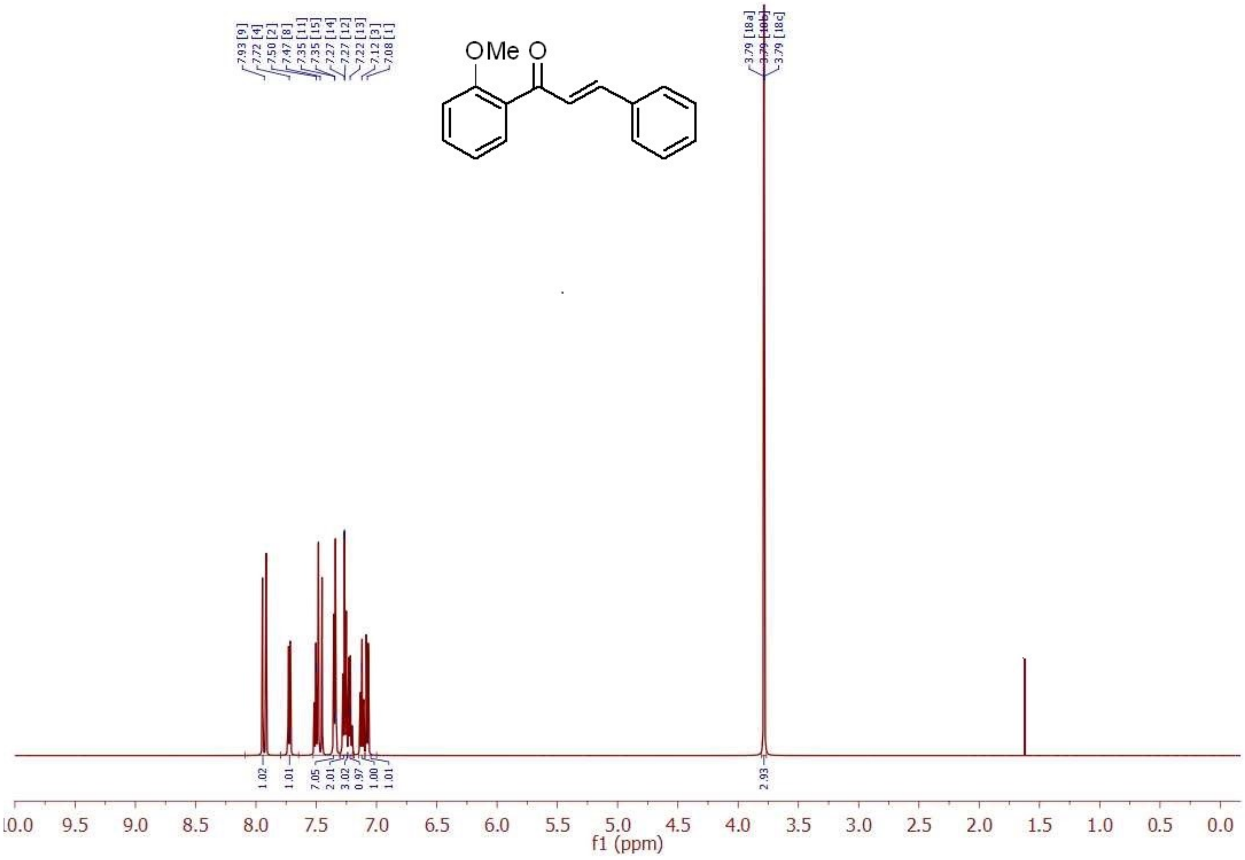

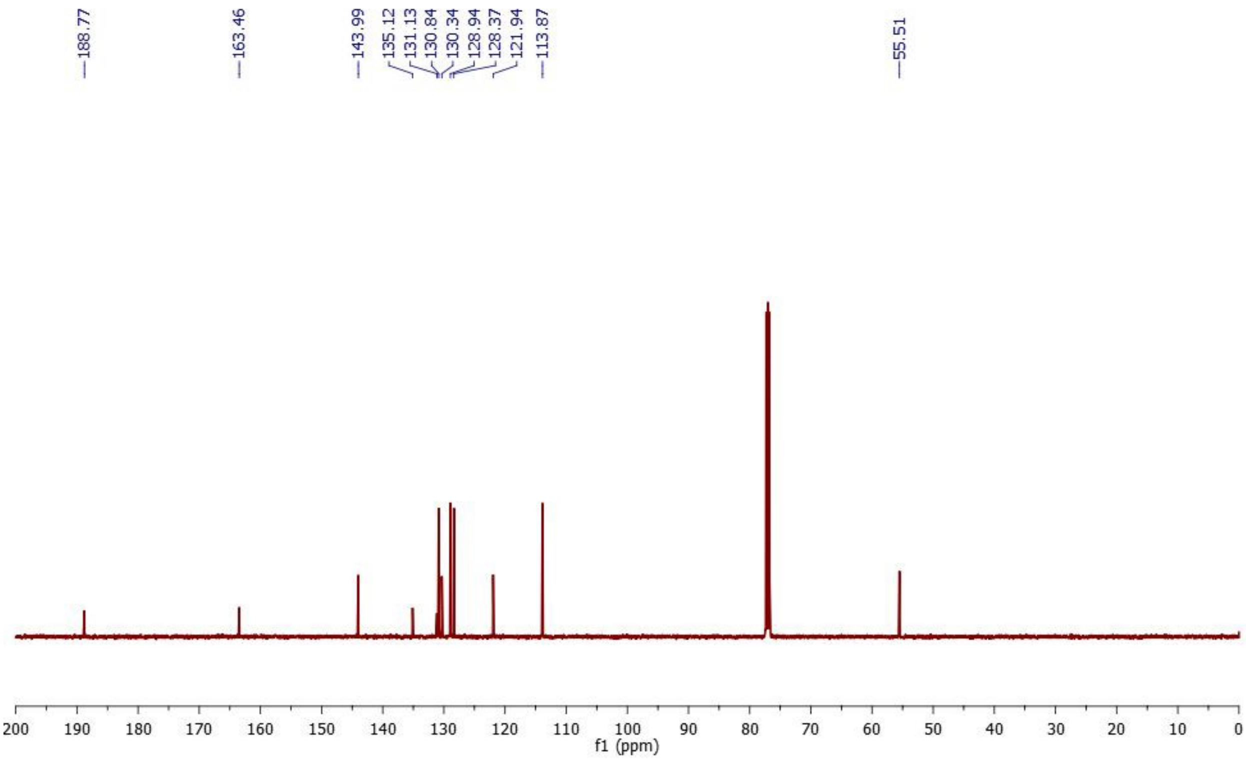

Fig.S27 ^{1}H and ^{13}C-NMR spectrum of 3bg (126 MHz, $CDCl_3$)

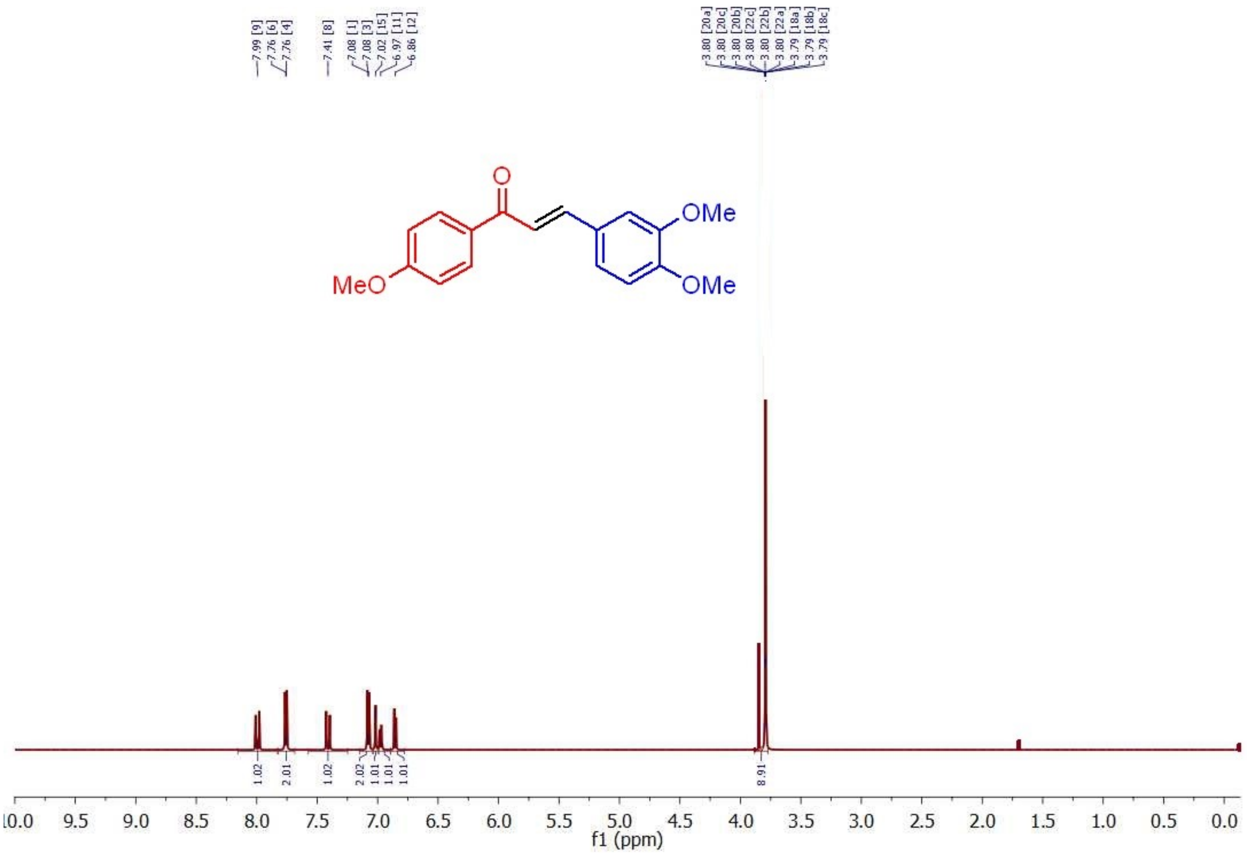

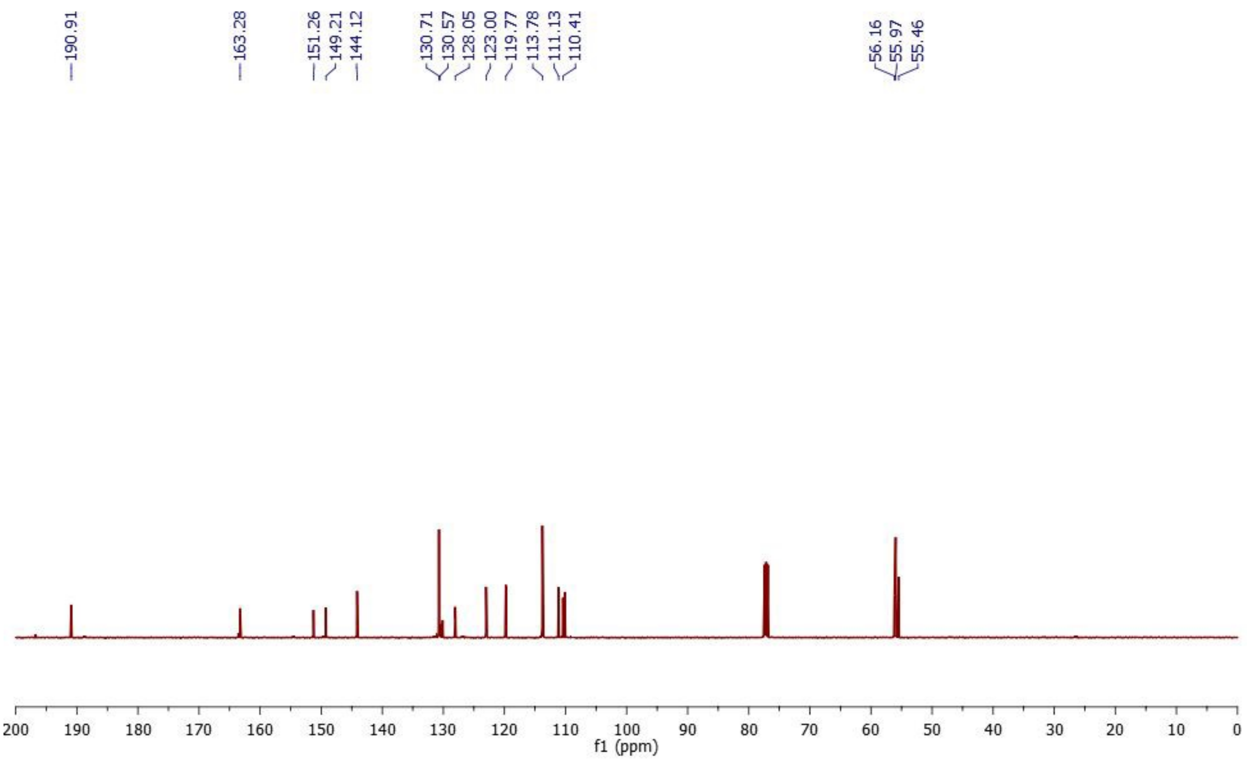

Fig.S28 ^{1}H and ^{13}C-NMR spectrum of 3ad (126 MHz, $CDCl_3$)

5. Additional Information HRMS Spectrum of reaction intermediate (A) and (B)

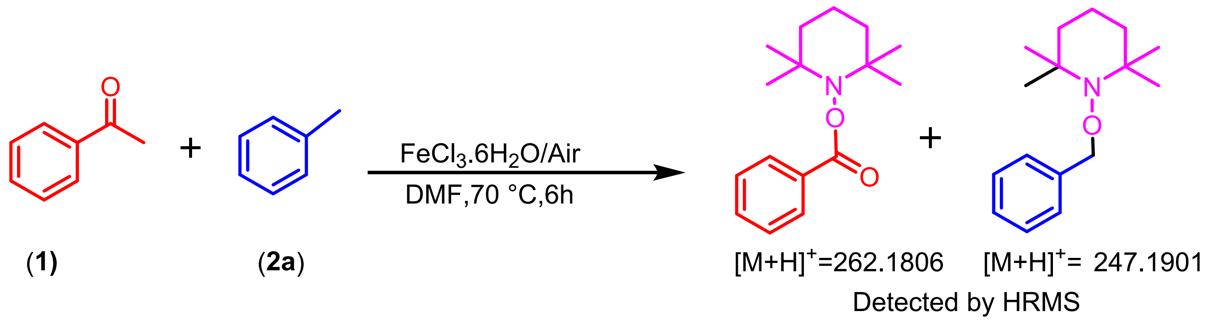

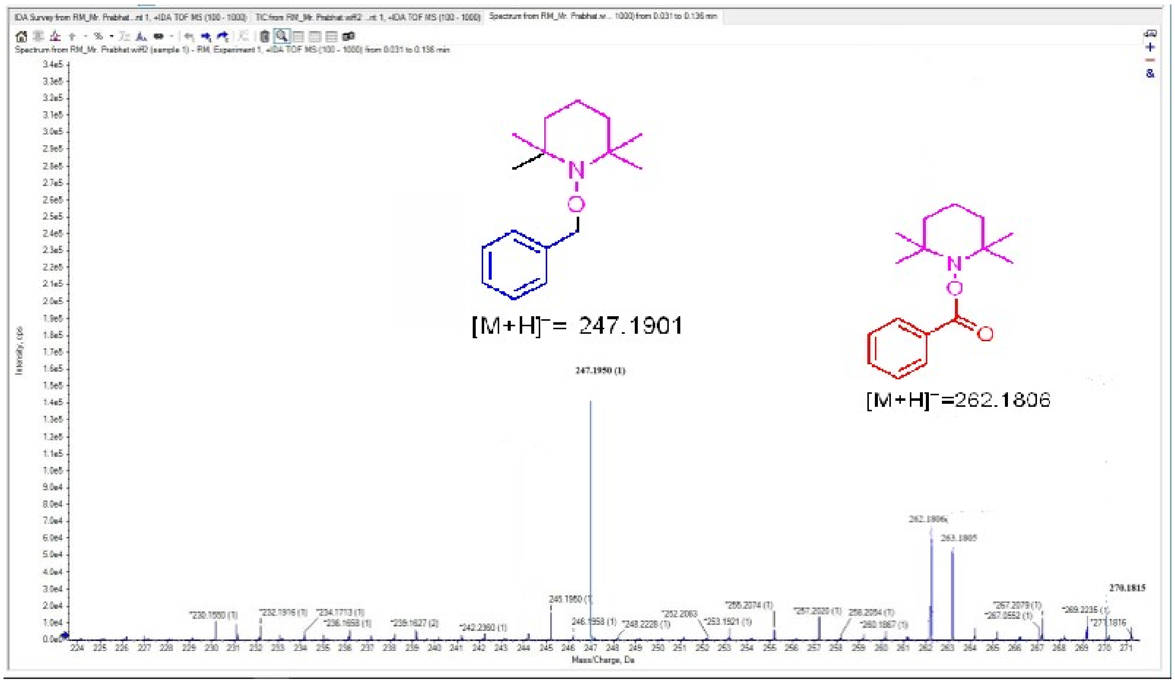

Fig.S29: HRMS of reaction mixture

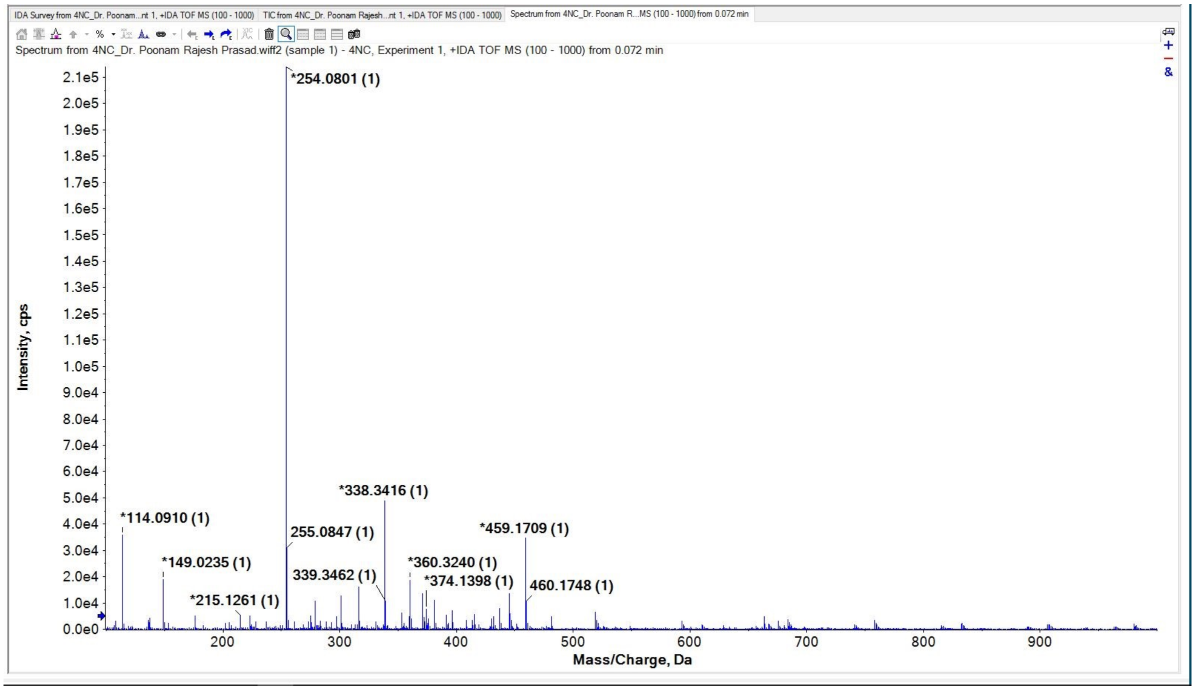

Fig.S30: HRMS of spectrum of 3d

Unsuccessful substrates scope

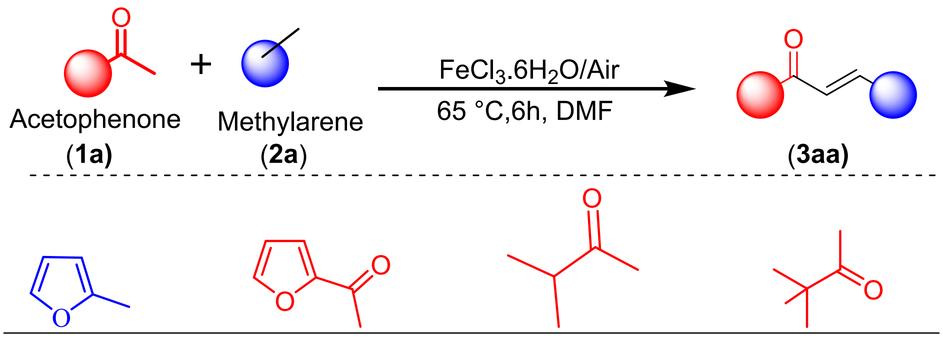

Fig.S31: List of Unsuccessful substrates

Aliphatic acetophenone exhibited poor reactivity among the tested substrates, resulting unsatisfactory yields, and the isolation of products were not achieved. Similarly, when 2-methyl furfural were employed, the yield was unsatisfactory, and product isolation was not successful.

6. References

1. Kumar, S. and Ahmed, N. *Green Chem.,*2016, *18*(**3**),.648-656.

2.Tripathi, S.; Kapoor, R.; Yadav, L. D. S., *Adv Synth &Catal.* 2018**,** *360* (**7**), 1407-1413.

3.Yadav, P., Yadav, M., Gaur, R., Gupta, R., Arora, G., Rana, P., Srivastava, A. and Sharma, R.K., *ChemCatChem*, 2020, *12*(**9**), 2488-2496.

4. Halpani, C. G.; Mishra, S.,*Tetrahedron Lett.* 2020**,** 61 (**31**), 152175.

5. Zhu, G.; Duan, Z.-C.; Zhu, H.; Qi, M.; Wang, D., *Mol Catal.* 2021**, *505***, 111-516.

6. Attar, S. R.; Sapkal, A. C.; Bagade, C. S.; Mujawar, S. H.; Kamble, S. B., *Mol Catal.,* 2023**, *542***, 113-120.

7. Elamathi, P., Chandrasekar, G. and Balamurali, M.M., *J. Porous Mater*., 2020. *27*(**3**), 817-829.

8. Deng, X.-Z.; Chen, Z.-Y.; Song, Y.; Xue, F.; Yamane, M.; Yue, Y.-N., *J. Org. Chem.,*2021**,** 86 (**18**), 12693-12704.

9. Dandia, A.; Saini, P.; Chithra, M.; Vennapusa, S. R.; Parewa, V., *J. Mol. Liq* 2021**, *331***, 115-758.

10. Qi, H.; Bai, Y.; Ren, Y.; Yuan, X.; Chen, S., *ChemistrySelect* **2023,** *8* (**17**), 010-04.
